# Transcriptional profiling of human cartilage endplate cells identifies novel genes and cell clusters underlying degenerated and non-degenerated phenotypes

**DOI:** 10.1186/s13075-023-03220-6

**Published:** 2024-01-03

**Authors:** Kyle Kuchynsky, Patrick Stevens, Amy Hite, William Xie, Khady Diop, Shirley Tang, Maciej Pietrzak, Safdar Khan, Benjamin Walter, Devina Purmessur

**Affiliations:** 1 Department of Biomedical Engineering, The Ohio State University, 3016 Fontana Laboratories, 140 W. 19th Ave, Columbus, OH 43210 USA; 2 Department of Biomedical Informatics, The Ohio State University, Columbus, OH USA; 3 Department of Chemistry and Biochemistry, The Ohio State University, Columbus, OH USA; 4 The James Comprehensive Cancer Center, The Ohio State University, Columbus, OH USA; 5 Department of Orthopaedics, The Ohio State University Wexner Medical Center, Columbus, OH USA

**Keywords:** Cartilage endplate, Intervertebral disc, Human, Degeneration, Bulk RNA-Seq, Single-cell RNA-Seq

## Abstract

**Background:**

Low back pain is a leading cause of disability worldwide and is frequently attributed to intervertebral disc (IVD) degeneration. Though the contributions of the adjacent cartilage endplates (CEP) to IVD degeneration are well documented, the phenotype and functions of the resident CEP cells are critically understudied. To better characterize CEP cell phenotype and possible mechanisms of CEP degeneration, bulk and single-cell RNA sequencing of non-degenerated and degenerated CEP cells were performed.

**Methods:**

Human lumbar CEP cells from degenerated (Thompson grade ≥ 4) and non-degenerated (Thompson grade ≤ 2) discs were expanded for bulk (*N*=4 non-degenerated, *N*=4 degenerated) and single-cell (*N*=1 non-degenerated, *N*=1 degenerated) RNA sequencing. Genes identified from bulk RNA sequencing were categorized by function and their expression in non-degenerated and degenerated CEP cells were compared. A PubMed literature review was also performed to determine which genes were previously identified and studied in the CEP, IVD, and other cartilaginous tissues. For single-cell RNA sequencing, different cell clusters were resolved using unsupervised clustering and functional annotation. Differential gene expression analysis and Gene Ontology, respectively, were used to compare gene expression and functional enrichment between cell clusters, as well as between non-degenerated and degenerated CEP samples.

**Results:**

Bulk RNA sequencing revealed 38 genes were significantly upregulated and 15 genes were significantly downregulated in degenerated CEP cells relative to non-degenerated cells (|fold change| ≥ 1.5). Of these, only 2 genes were previously studied in CEP cells, and 31 were previously studied in the IVD and other cartilaginous tissues. Single-cell RNA sequencing revealed 11 unique cell clusters, including multiple chondrocyte and progenitor subpopulations with distinct gene expression and functional profiles. Analysis of genes in the bulk RNA sequencing dataset showed that progenitor cell clusters from both samples were enriched in “non-degenerated” genes but not “degenerated” genes. For both bulk- and single-cell analyses, gene expression and pathway enrichment analyses highlighted several pathways that may regulate CEP degeneration, including transcriptional regulation, translational regulation, intracellular transport, and mitochondrial dysfunction.

**Conclusions:**

This thorough analysis using RNA sequencing methods highlighted numerous differences between non-degenerated and degenerated CEP cells, the phenotypic heterogeneity of CEP cells, and several pathways of interest that may be relevant in CEP degeneration.

**Supplementary Information:**

The online version contains supplementary material available at 10.1186/s13075-023-03220-6.

## Introduction

Low back pain is a common and potentially debilitating musculoskeletal disorder. It has been a leading cause of disability worldwide for decades [1] and is predicted to affect up to 80% of individuals in their lifetime [2] and afflict ~20% of older adults annually [3]. Cases relating to back pain are estimated to cost over $100 billion annually in the USA alone due to lost wages and medical expenses [4]. The socioeconomic challenges presented by back pain are projected to increase overtime [5]; thus, it is imperative to elucidate the causes of back pain and to improve therapeutics and preventative measures. Many cases of back pain originate from degenerated intervertebral discs (IVD) [6–8]. The IVDs are complex fibrocartilaginous pads that consist of a central gelatinous nucleus pulposus (NP) encapsulated peripherally by concentric collagen rings of the annulus fibrosus (AF) and axially by thin cartilage endplates (CEP). The healthy IVD provides pain-free flexibility to the spine, but it is susceptible to degeneration characterized by a loss of tissue integrity that decreases IVD height, alters IVD mechanics, and facilitates the invasion and sensitization of blood vessels, nerves, and immune cells that generate pain [9–13].

IVD degeneration is associated with a combination of external, lifestyle, and genetic factors [14], with one such factor being injury or degeneration of the CEPs [15]. Structurally, the CEPs encapsulate the NP to separate it from the subchondral bone and maintain a high internal pressure responsible for the disc’s mechanical function [15]. CEP defects can depressurize the NP and are often associated with altered disc mechanics that may initiate IVD degeneration [9, 16, 17]. CEP injury is also a strong predictor of pain [18], possibly by facilitating neurovascular ingrowth [10, 13, 19] and direct interactions between the NP and extradiscal tissue that can cause painful spinal cord sensitization [11] or Modic changes [20, 21]. The CEPs are also the primary route for nutrient transport to sustain the cells of the central region of the disc. Reductions in CEP permeability caused by increased calcification and other matrix compositional changes can disrupt nutrient supply to the central region of the disc and are associated with cell death and increased risk of IVD degeneration [22–24]; this could also complicate the efficacy of regenerative therapies for IVD degeneration that target the NP [25]. Therefore, it is evident that CEP degeneration can have a meaningful impact on the progression of IVD degeneration and back pain.

The phenotype and functions of the resident CEP cells and how these change during degeneration are less well characterized. Healthy CEP cells can regulate the tissue extracellular matrix by secretion of collagen II and proteoglycans [26] but upregulate catabolic enzymes during degeneration [27, 28]; loss of CEP cellularity is also associated with matrix degradation in vivo [29]. Degenerated CEP cells also upregulate inflammatory cytokines and may contribute to tissue calcification via the expression of hypertrophic and osteogenic markers [27, 30–33]. These cells are reminiscent of articular chondrocytes [34], and many studies assess the degenerative status of CEP cells using common markers associated with cartilage health and disease [27, 32, 35]; additionally, several mechanistic studies exploring non-traditional markers in CEP cells are motivated by previous findings in articular cartilage [36, 37]. Despite their similarities, the CEP and articular cartilage are structurally and compositionally distinct tissues [38], and analysis of a subset of cartilage and disc genes revealed phenotypic differences between the two cell types [34]. Taken together, CEP cells should be treated as a distinct cell type that should be better characterized. Additionally, the extent to which CEP cell phenotype shifts during degeneration is not well characterized.

This study aimed to thoroughly characterize the phenotypes of human CEP cells from degenerated and non-degenerated IVD tissue using bulk RNA sequencing followed by single-cell RNA sequencing. First, we used bulk RNA sequencing to compare the broad gene expression of non-degenerated and degenerated human CEP cells and performed a literature search to determine the novelty of the genes from our dataset in CEP cells and other cartilaginous tissues. Next, we used single-cell RNA sequencing to identify and characterize different subpopulations of human CEP cells and to study their expression of the genes in our bulk RNA sequencing dataset. We hypothesized that non-degenerated and degenerated human CEP cells have distinct gene expression profiles and that we will identify numerous genes that were previously unstudied in CEP cells. We also hypothesized that both non-degenerated and degenerated CEP cell samples will consist of numerous cell subpopulations with distinct gene expression and functional profiles. The results of this study may be used to identify novel differences between non-degenerated and degenerated CEP cells and open the door to identifying novel CEP cell-specific markers.

## Methods

All reagents were purchased from Thermo Fisher Scientific (Waltham, MA, USA) or VWR International (Radnor, PA, USA) unless otherwise stated.

### Cell isolation

Primary cells from de-identified human cadaveric CEP tissue were used for all experiments. Tissue was harvested from cadaveric lumbar spines obtained through Ohio State University’s Cooperative Human Tissue Network under an Institutional Review Board exemption within 48 h of death as described previously [32]. Spines were cleaned of soft tissue and dissected to isolate individual vertebra-disc-vertebra motion segments, which were then sectioned in the sagittal plane and imaged for Thompson grading by two independent investigators. After, the IVDs were separated from the adjacent vertebrae, exposing the CEP tissue. The CEP was carefully scraped away from the vertebrae using a scalpel blade, cut into smaller pieces, and digested sequentially with 0.2% w/v protease from *Streptomyces griseus* (Cat#P5147; Sigma-Aldrich, St. Louis, MO, USA) for 1 h and 0.2% w/v collagenase II from *Clostridium histolyticum* (Cat#17101015) for 4 h. The digests were passed through 70-μm strainers and expanded in standard media (high-glucose Dulbecco’s modified Eagle medium (DMEM), 10% fetal bovine serum (FBS), 100 U/mL penicillin/streptomycin, 0.5% fungizone, 50 μg/mL freshly added ascorbic acid) and incubated at 5% CO_2_, 21% O_2_, 37 °C for at least one passage. The cells were frozen at − 80 °C in 10% dimethyl sulfoxide in FBS until needed for experiments. CEP cells used in experiments were classified as “non-degenerated” if from an IVD joint of grade ≤ 2 or “degenerated” if from an IVD joint of grade ≥ 4; sample demographics for bulk and single-cell RNA sequencing are provided in Table 1 and Table 2, respectively.

**Table 1:** Sample demographics for bulk RNA-Seq

Sample ID	Group	Level	Age	Sex	Thompson grade
Hu12	Non-degenerated	L2/L3	31	Female	II
Hu16	Non-degenerated	L4/L5	19	Female	II
Hu19	Non-degenerated	L4/L5	33	Male	II
Hu21	Non-degenerated	L3/L4	40	Female	II
Hu15	Degenerated	L2/L3	59	Female	IV
Hu17	Degenerated	L3/L4	39	Male	IV
Hu18	Degenerated	L3/L4	74	Male	IV
Hu23	Degenerated	L4/L5	42	Female	IV

**Table 2:** Sample demographics for single-cell RNA-Seq

Sample ID	Group	Level	Age	Sex	Thompson grade
Hu21	Non-degenerated	L3/L4	40	Female	II
Hu23	Degenerated	L3/L4	42	Female	IV

### Bulk RNA sequencing sample preparation and data analysis

For bulk RNA sequencing, CEP cells (passage ≤ 2) from non-degenerated (*N*=4) and degenerated (*N*=4) IVDs were thawed, plated in T75 flasks at a density of 0.5–1 × 10^6^ cells per flask, and expanded until ~80% confluent. During culture, cells were kept at 37 °C, 21% O_2_, and 5% CO_2_ and were fed 2–3 times per week with standard media. After expansion, the cells were washed once with 1X PBS and collected in TRIzol reagent (Cat#15596026). RNA was isolated using a PureLink RNA MINI kit (Cat#12183018A), and a preliminary assessment of RNA concentration and purity (260/280 and 260/230 ratios) was performed using a NanoDrop 2000c spectrophotometer. To assess suitability of total RNA for bulk RNA sequencing, RNA Integrity Number (RIN) was measured using BioAnalyzer RNA Nano Kit (Cat#5067-1511; Agilent Technologies, Inc., Santa Clara, CA, USA), and the RNA concentration was fluorometrically quantified using Qubit RNA High Sensitivity Assay (Cat#Q32852).

Samples with RIN >7 were used to generate mRNA sequencing libraries using a NEBNext® Ultra™ II Directional (stranded) RNA Library Prep Kit (Cat#E7760L; New England BioLabs, Inc., Ipswich, MA, USA) and a NEBNext Poly (A) mRNA Magnetic Isolation Module (Cat#E7490; New England BioLabs, Inc.). Briefly, 200 ng of total RNA were used as sample input. RNA fragmentation was set at 10 min, and 12 polymerase chain reaction (PCR) cycles were used in final library generation. Library quantification and characterization were assessed with a BioAnalyzer High Sensitivity DNA Kit (Cat#5067-4626; Agilent Technologies, Inc.) and a Qubit DNA High Sensitivity Assay Kit (Cat#Q32854). Libraries were pooled together with other index-compatible RNA sequencing libraries for sequencing on a NovaSeq 6000 (Illumina, San Diego, CA, USA) paired-end 100bp flow cell to a minimum depth of 20 million clusters per sample.

**Fig. 1:**
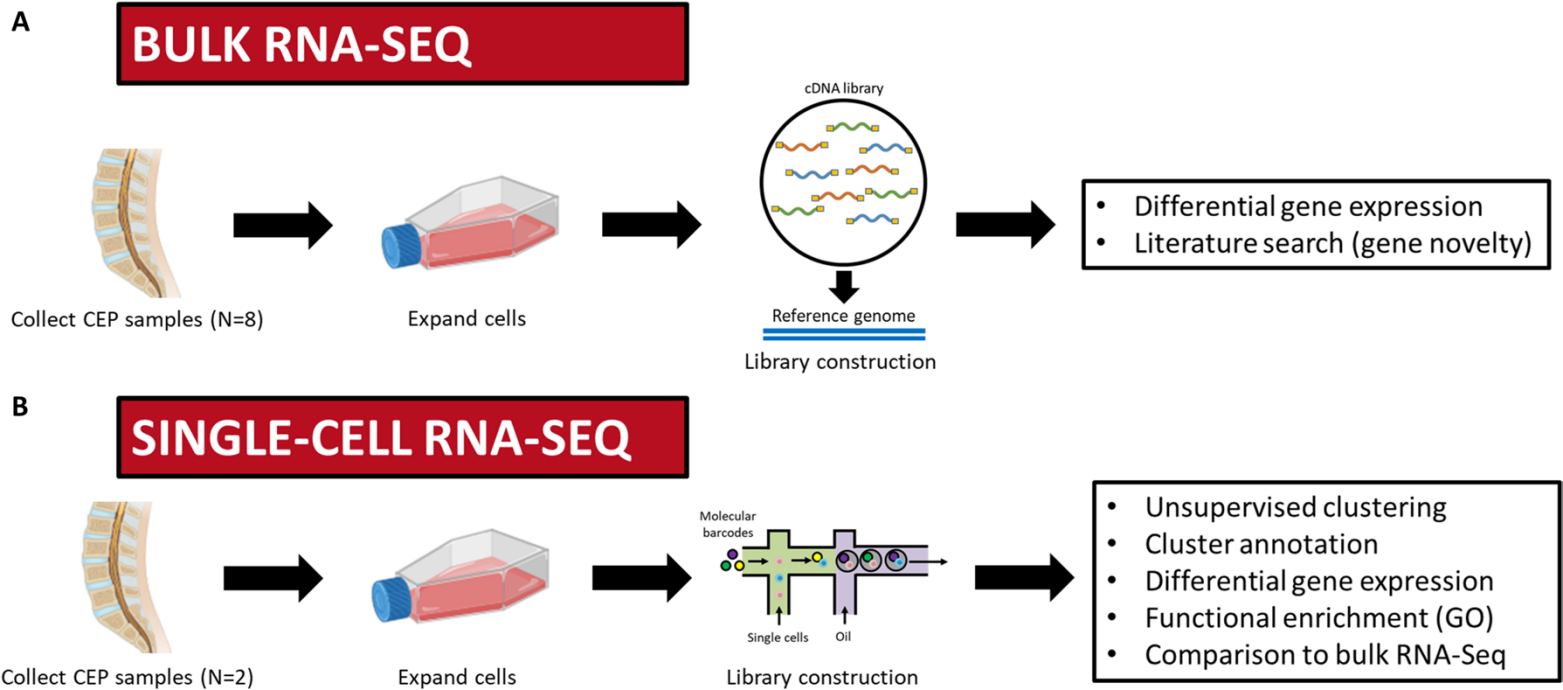
Conceptual workflow of RNA sequencing experiments. For both bulk RNA sequencing (**A**) and single-cell RNA sequencing (**B**), CEP cells from human cadaveric lumbar spines were isolated, expanded, and processed. A list of analyses performed within each dataset are also listed. Figure created with licensed Biorender.com software

Basic analysis was performed using a custom in-house pipeline in which raw FASTQ data were aligned to the human reference genome GRCh38 using hisat2 v2.1.0 [39, 40]. Gene-wise counts were generated with featureCounts from the subread package v1.5.1 [41] for genes annotated by ensemble Homo_sapiens.GRCh38.101, counting the primary alignment in the case of multimapped reads. Differential expression analysis was performed using deseq2 [42] and included batch as a covariate to correct for batch biases internally. An rlog normalization of the counts was performed to visualize the results, and a batch correction using removeBatchEffect from limma was performed [43]. A conceptual overview of our bulk RNA sequencing experiment is provided in Fig. 1A.

### Single-cell RNA sequencing sample preparation and data analysis

Passage 1 non-degenerated (*N*=1) and degenerated (*N*=1) CEP cells were thawed, plated in T75 flasks at a density of 0.5–1 × 10^6^ cells per flask, and expanded until ~80% confluent. During culture, cells were kept at 37 °C, 21% O_2_, and 5% CO_2_ and were fed 2–3 times per week with standard media. After expansion, the cells were processed according to 10X Genomics instructions [44]. Briefly, the cells were put into suspension, filtered using a 70-μm strainer, rinsed three times with 1X PBS containing 0.04% bovine serum albumin (BSA) (Cat#A9418; Sigma-Aldrich), and re-suspended at a concentration of 700–1200 cells/μL in 1X PBS containing 0.04% BSA.

Single-cell suspensions were processed using a Chromium Next GEM Single Cell 3’ Kit v3.1 (PN-1000268, 10X Genomics, Pleastanton, CA) according to manufacturer’s instructions [45]. For each sample, ~16,500 cells were loaded onto a Chromium Next GEM Chip G (PN-2000177, 10X Genomics) to prepare barcoded single-cell gel beads-in-emulsion (GEMs) with a targeted cell recovery of ~10,000 cells. Barcoded cells in GEMs were lysed and cDNA was synthesized and amplified for 11 cycles via PCR. Quality control for cDNA yield and quality was performed using an Agilent 2100 Bioanalyzer at a 1:10 cDNA dilution; samples had final cDNA concentrations between 48 and 54 ng/μL in 40 μL of buffer (1920–2160 ng total cDNA). For each sample, library construction with 25% of the total cDNA content (~475 ng) was started by fragmenting and end-repairing the sample, then amplifying for 10–12 cycles using PCR. Quality control was repeated on the cDNA fragments using a Bioanalyzer High Sensitivity chip (Agilent Technologies, Inc.) prior to sequencing. Samples were sequenced using a NovaSeq 6000 SP flow cell 100-cycle kit (Illumina) in a Read1:i7:i5:Read2 of 28:10:10:90 bp format according to manufacturer’s recommendations [45] to produce 100-bp reads for subsequent analysis. In all, 8035 non-degenerated cells were sequenced at a depth of ~28,000 reads per cell and 9122 degenerated cells were sequenced at a depth of ~24,000 reads per cell, both with a mapping rate of 98%.

Sequenced single cell reads in FASTQ format were aligned to a reference transcriptome using the human genome in 10X Genomics Cell Ranger software (Cell Ranger 6.1.2) [46] based on default parameters with include-introns function. Output files from CellRanger count were analyzed using Seurat 4.0.4 [47]. Cells expressing 200–7000 unique genes and containing < 10% mitochondrial gene content were selected for further analysis. An averaged expression matrix from each cluster was exported from Seurat using the AverageExpression function, and gene expression values were normalized using a *z*-score calculation function and plotted using pheatmap function [48]. Gene Ontology (GO) functional enrichment analysis was performed using clusterProfiler 4.6.0 toolkit based on default settings [49]. A conceptual overview of our single-cell RNA sequencing experiment is provided in Fig. 1B.

### Bulk RNA sequencing literature review

A PubMed literature review was performed to determine which significant genes from the current bulk RNA sequencing dataset were previously identified and studied in different cartilaginous tissues (CEP, IVD, cartilage). The PubMed database was accessed on April 15, 2023, using the National Library of Medicine’s Entrez Programming Utilities following their data extraction guidelines [50]. Searches were conducted by combining relevant search terms for one tissue and one gene at a time. For each gene, the search terms consisted of the gene symbol and the full gene name from genecards.org. For CEP searches, the search terms included “cartilage endplate”, “endplate”, “endplate chondrocyte”, and “cartilaginous”. For IVD searches, the search terms were “IVD”, “intervertebral disc”, “intervertebral disk”, “degenerative disc disease”, “degenerative disk disease”, “nucleus pulposus”, and “annulus fibrosus”. For miscellaneous cartilage searches, the search terms were “chondrocyte”, “cartilage”, “growth plate”, “articular”, “arthritis”, “osteoarthritis”, and “rheumatoid arthritis”. Results were manually reviewed to exclude papers in which the tissue or the gene-of-interest was not the subject of the study; transcriptomic studies, proteomic studies, and review articles that identified or discussed a gene of interest in the tissue of interest were not excluded. The PubMed IDs for all identified papers until April 15, 2023, were saved and the final number of genes with or without prior publications in each tissue of interest were recorded (Table S1).

## Results

### Bulk RNA sequencing

#### Numerous genes were differentially expressed between non-degenerated and degenerated CEP samples

**Fig. 2:**
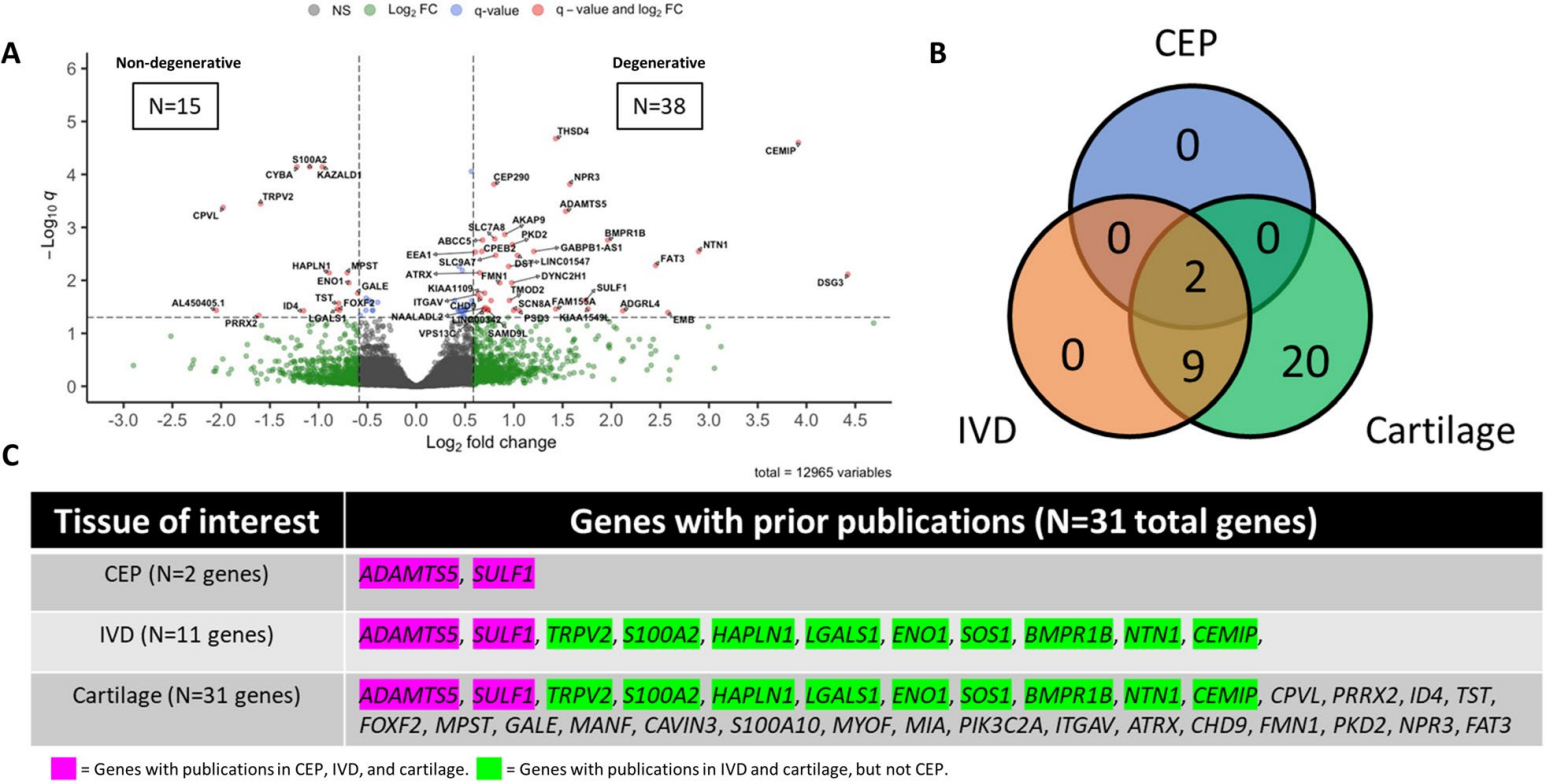
Bulk RNA sequencing results. **A** The breakdown of D.E. genes between non-degenerated and degenerated CEP samples is depicted by a volcano plot. Positive F.C. indicates upregulation in degenerated samples, whereas negative F.C. indicates upregulation in non-degenerated samples. Thresholds for upregulation were set at |F.C.| ≥ 1.5 and significance was set at *q* < 0.05. Boxed numbers indicate number of significantly D.E. genes in each compartment of plot. **B** The results of the literature review are summarized by a Venn diagram showing the number of genes previously studied in the CEP, the IVD, or cartilage. **C** Breakdown of the genes previously studied in each tissue of interest. Purple-highlighted genes were previously studied in all three tissues and green-highlighted genes were previously studied in the IVD and cartilage only. F.C., fold change; D.E., differentially expressed

We first constructed gene libraries for human CEP cells from non-degenerated and degenerated IVDs using bulk RNA sequencing. 12,965 genes were detected across all samples, of which 76 were significantly differentially expressed (D.E.) between non-degenerated and degenerated CEP cells (*q* < 0.05). 15 genes were upregulated in non-degenerated CEP cells (fold change ≤ − 1.5), and 38 genes were upregulated in degenerated CEP cells (fold change ≥ + 1.5) (Fig. 2A); 23 additional genes were D.E. but with a fold change magnitude less than 1.5. The fold change and *q*-values of all significantly D.E. genes are provided in Table S2. The genes in our dataset encoded for proteins that support a broad array of functions, including signaling, molecular transport, transcriptional and translational regulation, metabolism and cell cycling, cytoskeletal and matrix regulation, protein binding and modification, and inflammation. Notably, *GALE* and *HAPLN1*, markers involved in extracellular matrix (ECM) synthesis and stabilization [51, 52], were upregulated in non-degenerated CEP cells; conversely, *ADAMTS5* and *CEMIP*, enzymes that break down ECM molecules [28, 53], were upregulated in degenerated CEP cells. This was an expected trend, as excessive matrix breakdown via decreased matrix synthesis and increased MMP and aggrecanase activity is a hallmark of cartilage disease [54, 55].

Among the remaining genes, many associated with metabolism and the cell cycle were upregulated in non-degenerated cells, whereas many associated with molecular transport, transcriptional and translational regulation, and cytoskeletal and matrix regulation were upregulated in degenerated cells (Table 3). Specifically, non-degenerated cells were enriched in genes that may be implicated in chondrocyte differentiation and skeletogenesis (*FOFX2*, *PRRX2*, *S100A2*, *ID4*, *KAZALD1*), metabolism (*ENO1*, *TST*, *MPST*), matrix production and adhesion (*GALE*, *HAPLN1*, *LGALS1*), and immunogenic functions (*CPVL*, *CYBA*). Also enriched in non-degenerated cells were *TRPV2*, an ion channel that is downregulated in aging cartilage and is necessary for chondrocyte mechanotransduction [56], and *AL450405.1*, a pseudogene that may be associated with ribosome biogenesis. Several of these genes (*MPST*, *TRPV2*) were reported to protect against cartilage calcification in in vivo models of surgically induced osteoarthritis [56, 57].

**Table 3:** Breakdown of differentially expressed genes with *q* < 0.05, |fold change| ≥ 1.5 (*N* = 53 total genes)

Category	Genes
Ligand/receptor binding, signaling, chemotaxis	**Non-degenerated**: *KAZALD1* **Degenerated**: *BMPR1B*, *NTN1*, *NPR3*, *SAMD9L*
Signal transduction (GPRC, 2nd messengers, etc.)	**Non-degenerated**: ---- **Degenerated**: *ADGRL4*, *PSD3*
Intracellular and/or membrane transport	**Non-degenerated**: *TRPV2* **Degenerated**: *FAM155A*, *SCN8A*, *SLC7A8*, *SLC9A7*, *EEA1*, *VPS13C*, *DYNC2H1*, *KIAA1109*, *ABCC5*, *PKD2*, *CEP290*
Transcription, RNA-binding, and translation regulation	**Non-degenerated**: *FOXF2* **Degenerated**: *KLF12*, *CPEB2*, *GABPB1-AS1*, *LINC01547*, *LINC00342*, *ATRX*, *CHD9*
Metabolism, proliferation, differentiation, and cell cycle	**Non-degenerated**: *ENO1*, *PRRX2*, *TST*, *MPST*, *GALE*, *S100A2*, *ID4* **Degenerated**: ----
Cytoskeleton, ECM, and cell/matrix adhesion	**Non-degenerated**: *HAPLN1*, *LGALS1* **Degenerated**: *DSG3*, *THSD4*, *DST*, *TMOD2*, *ADAMTS5*, *CEMIP*, *ITGAV*, *FMN1*, *FAT3*, *EMB*, *AKAP9*
Proteolysis, protein-binding, and protein modification activity	**Non-degenerated**: *----* **Degenerated**: *SULF1*
Immunogenic, inflammatory, and stress function	**Non-degenerated**: *CYBA*, *CPVL* **Degenerated**: ----
Unclear function	**Non-degenerated**: AL450405.1 **Degenerated**: *KIAA1549L*, *NAALADL2*

Conversely, degenerated cells were enriched in genes associated with signaling and signal transduction (*BMPR1B*, *NTN1*, *NPR3*, *ADGRL4*, *PSD3*, *SAMD9L*, *PKD2*), endosomal pathways (*EEA1*, *VPS13C*, *SAMD9L*), intracellular transport (*CEP290*, *DYNC2H1*, *KIAA1109*), membrane transport (*PKD2*, *SCN8A*, *SLC9A7*, *SLC7A8*, *AKAP9*, *ABCC5*), cytoskeletal function (*TMOD2*, *DST*, *AKAP9*, *FMN1*), matrix and adhesion functions (*ADAMTS5*, *CEMIP*, *THSD4*, *ITGAV*, *FAT3*, *EMB*, *DSG3*), and transcriptional and translational regulation (*ATRX*, *CHD9*, *CPEB2*, *GABPB1-AS1*, *LINC00342*, *LINC01547*, *KLF12*). Two genes with unknown functions (*KIAA1159L*, *NAALADL2*) were also enriched in degenerated cells (Table 3).

#### Literature search: many differentially expressed genes have not been reported on in CEP cells

CEP cells are significantly understudied compared to their IVD cell counterparts; therefore, we conducted a PubMed literature review to determine whether the genes in our dataset have been previously studied in CEP cells. Of the 76 genes identified, only 2 (*ADAMTS5*, *SULF1*) have been studied previously in CEP cells (Fig. 2B). *ADAMTS5* is a major aggrecanase in cartilaginous tissues, is upregulated in the degenerated CEP, and is commonly used as a marker of CEP cell degeneration [28, 58]. *SULF1* is an enzyme that de-sulfonates heparan sulfated proteoglycans [59, 60], which regulate several signaling pathways such as FGF, Wnt, TGFβ, and hedgehog [60–62]; heparan sulfated proteoglycans are also a major constituent of the pericellular matrix proteoglycan perlecan [63]. However, *SULF1* was previously studied in a single global in vivo knockout model of disc degeneration [64], and its specific role in CEP cells has not been fully ascertained.

Twenty-nine additional genes were previously studied in cells from the IVD or other cartilaginous tissues, with most being exclusively studied in articular and growth plate chondrocytes (Fig. 2B, Fig. 2C). Interestingly, most genes upregulated in non-degenerated CEP cells were found in this category, and they were mostly associated with metabolic and cell cycling functions (Table S3). Forty-five genes whose relevance in cartilaginous cells has not been previously documented remained (Table S4). Many of these genes were upregulated in degenerated CEP cells and were associated with signaling, molecular transport, transcriptional and translational regulation, and cytoskeletal and matrix regulation. The trends in gene expression between non-degenerated and degenerated CEP cells may point to possible pathways that become dysregulated due to degeneration and may be future targets of investigation.

### Single-cell RNA sequencing

#### Numerous unique cell subpopulations were identified in non-degenerated and degenerated CEP samples

**Fig. 3:**
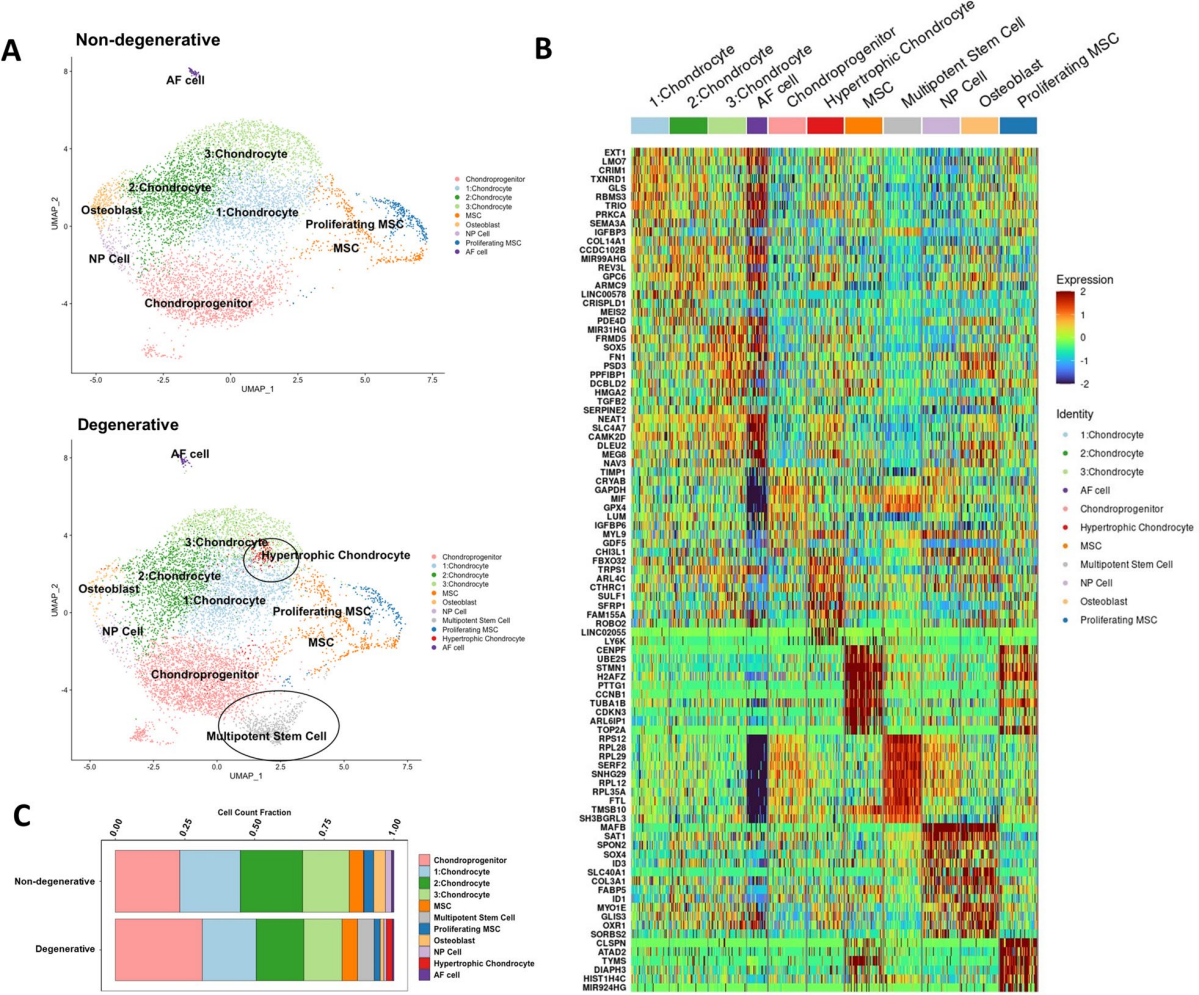
Single-cell RNA sequencing results. **A** Unsupervised clustering of non-degenerated (upper) and degenerated (lower) CEP samples reveals sample heterogeneity. Most clusters are shared between both samples, though hypertrophic chondrocyte and multipotent stem cell clusters were only present in the degenerated sample (black circles). Cluster annotations were determined from manually curated markers for different cell types predicted to reside within the CEP. **B** Heatmap of differentially expressed genes across all clusters. **C** A breakdown of the cellular composition of each sample, by percentage

We next performed single-cell RNA sequencing on human CEP cell samples isolated from one non-degenerated and one degenerated IVD joint. Quality control found low enrichment of mitochondrial, ribosomal, and hemoglobin, and platelet RNA, indicating good sample mRNA purity (Fig. S1). Unsupervised clustering identified 11 unique clusters across both samples (Fig. 3A), and differential gene expression analysis indicated that each cluster had a unique gene signature (Fig. 3B, Fig. S2). Clusters were annotated using markers for cell types predicted to reside within the CEP or to be co-isolated during tissue isolation (Table S5, Table S6). We identified 4 distinct chondrocyte clusters, 4 distinct stem cell clusters, an osteoblast cluster, an NP cell cluster, and an AF cell cluster (Fig. 3B). Both samples had a similar compositional breakdown, with chondroprogenitor cells and chondrocytes being the most plentiful cell types while NP cells, AF cells, and osteoblasts were least plentiful (Fig. 3C). Notably, multipotent stem cell and hypertrophic chondrocyte clusters were found exclusively in the degenerated sample. Multipotent stem cells are a class of progenitor cell that can differentiate into several cell types within a particular lineage and includes mesenchymal stem cells [65], though we were unable to resolve the specific cell type within this cluster. Hypertrophic chondrocytes are terminally mature chondrocytes that are upregulated in degenerated cartilaginous tissues and facilitate matrix degradation, matrix calcification, and vascular invasion as part of endochondral ossification [66–68].

#### Chondrocyte gene and functional enrichment profiles indicate cluster-specific functions

**Fig. 4:**
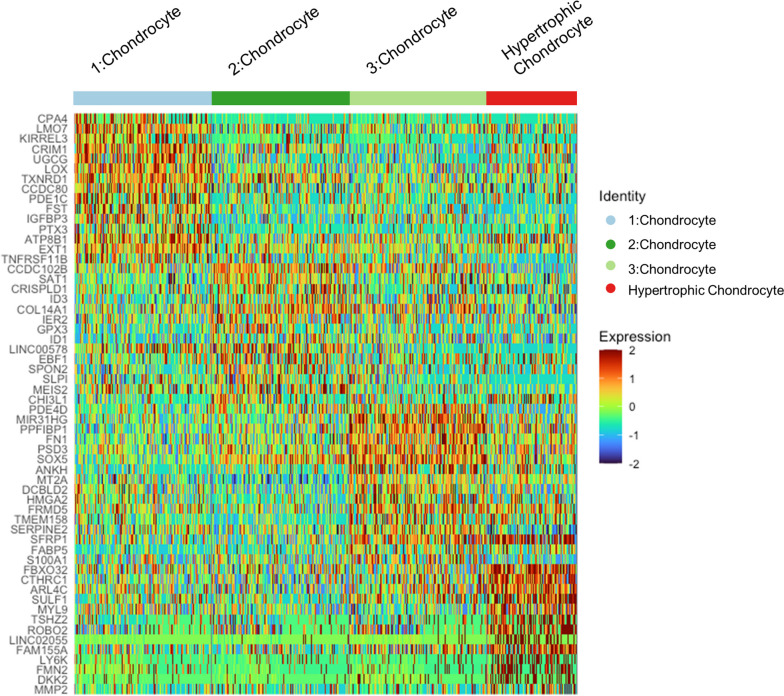
Heatmap of differentially expressed genes across all chondrocyte clusters. Cells from the non-degenerated and degenerated samples were pooled in this analysis

**Fig. 5:**
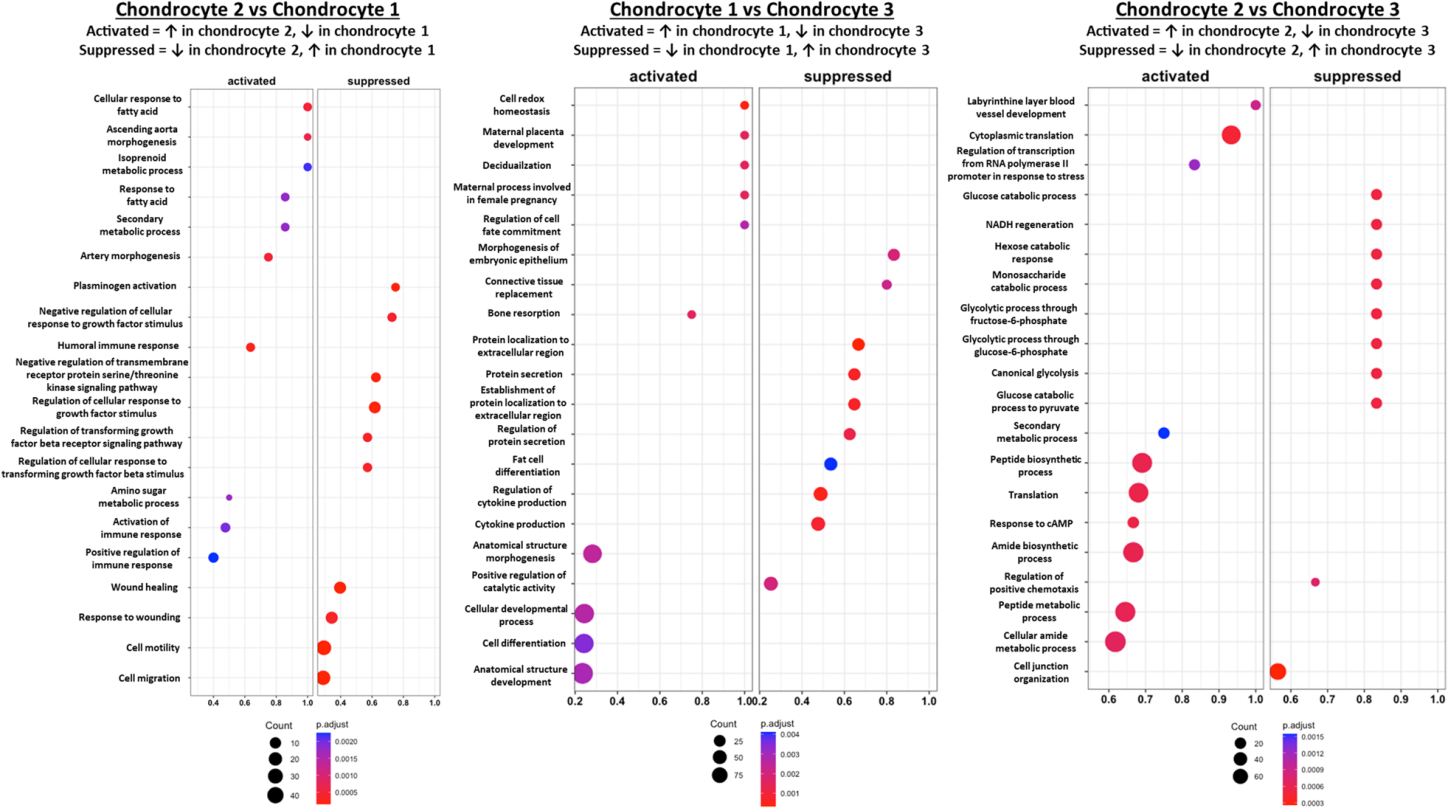
Gene ontology comparing functional enrichment of biological processes between different chondrocyte clusters. From left to right: chondrocyte 2 vs. chondrocyte 1, chondrocyte 1 vs. chondrocyte 3, chondrocyte 2 vs. chondrocyte 3. Cells from the non-degenerated and degenerated samples were pooled in this analysis

The 4 chondrocyte clusters in our dataset were phenotypically distinct from each other and appeared to have variable functions according to their top differentially expressed genes and GO functional enrichment (Fig. 4, Fig. 5). Whereas hypertrophic chondrocytes are a well-defined cell type, the functions of the other 3 chondrocyte clusters are not as immediately apparent.

Cells in the chondrocyte 1 cluster were enriched in pathways such as wound healing, tissue morphogenesis, growth factor stimulus, regulation of cell fate commitment, and cell migration. Wound healing and tissue morphogenesis involve regulation of various cytokine and growth factor pathways to properly coordinate cell migration, proliferation, and differentiation [69]. In support of this, this cluster was enriched in genes that directly or indirectly regulate various signaling pathways (*LMO7*, *CRIM1*, *FST*, *IGFBP3*, *PTX3*, *EXT1*), including the FGF, TGFβ superfamily, hedgehog, and IGF pathways [70–76]. Based on these observations, this cluster may have a prominent signal regulatory function.

Cells in the chondrocyte 2 cluster expressed genes that regulate stemness (*SAT1*, *EBF1*), mitotic activity (*CCDC102B*, *IER2*, *ID1*, *ID3*), or canonical Wnt signaling (*SPON2*), which regulates cell proliferation in many cell types [77–79]. They also upregulated *MEIS2*, which regulates chondrogenesis and commonly forms complexes with *Pbx1* [80, 81], a patterning factor that is co-expressed with *MEIS2* in developing cartilage and is upregulated in proliferating chondrocytes [80, 82]. However, chondrocyte 2 cells were not enriched in markers of G1/S or mitosis (Fig. S3, Fig. S4, Fig. S5, Fig. S6, Fig. S7). Instead, GO analysis showed an enrichment of immune regulation, protein translation, and various metabolic processes. It is possible this is a more quiescent, biosynthetic type of chondrocyte, but the current findings do not support a definitive role for this cluster.

Cells in the chondrocyte 3 cluster expressed genes regulating chondrogenesis and calcification, such as *SOX5* [83], *ANKH* [35, 84], *SFRP1* [85], *S100A1* [83, 86], and *HMGA2* [87]. They also expressed *PPFIBP1*, which may promote normal long bone development as it is expressed in osteoblasts and has been mapped to a chromosomal locus associated with defects that induce congenital limb shortening [88, 89]. Taken together, cells in this cluster may support a chondrogenic phenotype or may be transitioning to a degenerated pre-hypertrophic phenotype. In support of this, these cells were enriched for pathways related to connective tissue replacement, cytokine production, and positive regulation of chemotaxis, which aligns with the secretion of angiogenic and osteoclastogenic signals by hypertrophic chondrocytes during endochondral ossification to promote vascular and osteoclast invasion into degrading cartilage [68]. Genes associated with the regulation of glycolytic and sugar metabolism pathways were also enriched; this is notable as glycolysis and oxidative phosphorylation dynamics vary at different stages of chondrocyte differentiation [90].

#### Progenitor cell gene and functional enrichment profiles indicate cluster-specific functions

**Fig. 6:**
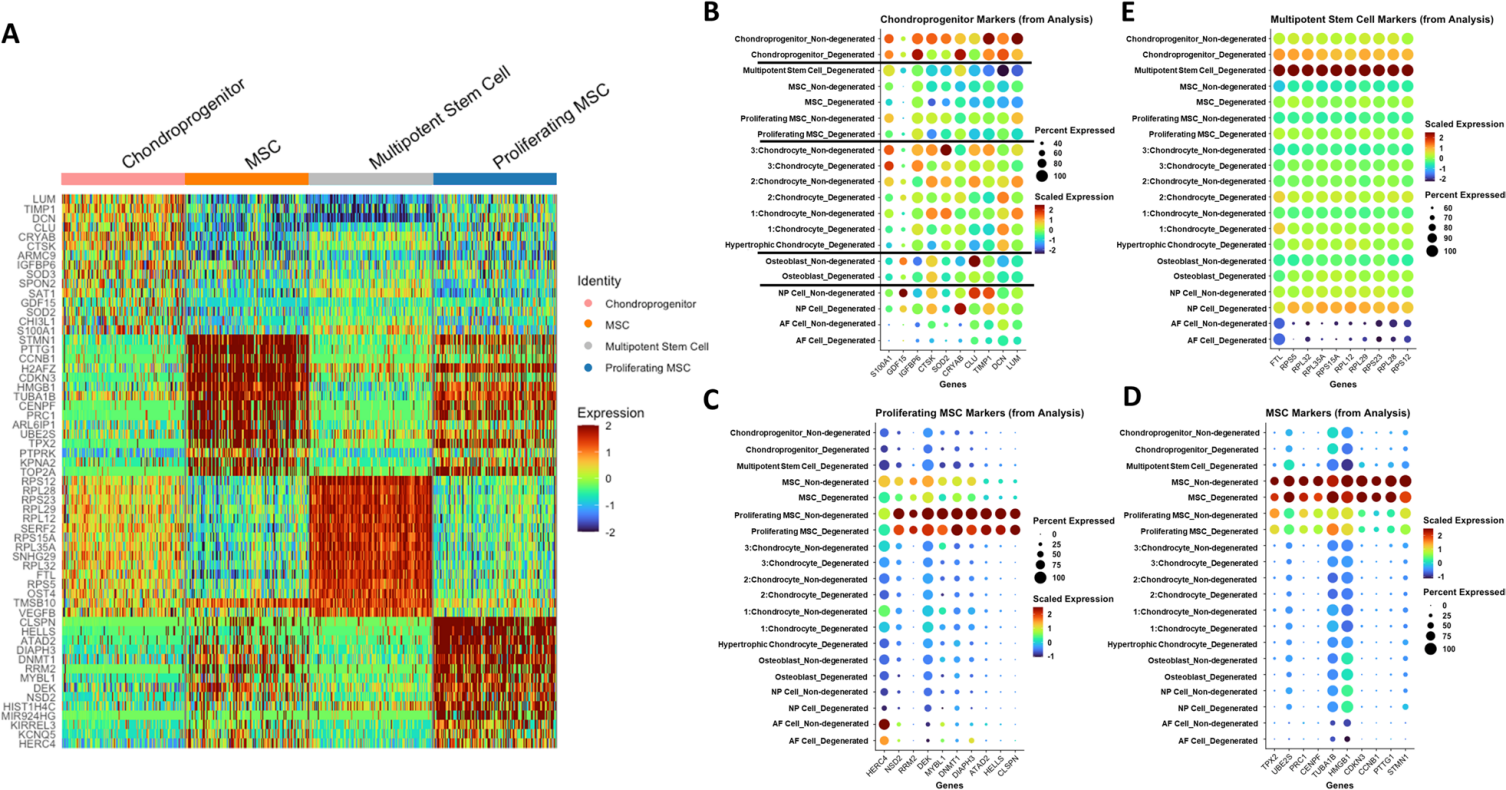
Comparison of progenitor cell clusters in all samples. **A** Heatmap of differentially expressed genes across all progenitor cell clusters. Cells from the non-degenerated and degenerated samples were pooled in this analysis. **B**–**E** Dot plots of top differentially expressed markers for **B** chondroprogenitor cells, **C** proliferating MSCs, **D** MSCs, and **E** multipotent stem cells from the current analysis. Black lines on **B** separate chondroprogenitors, MSCs, and chondrocyte clusters

We next characterized the different progenitor cell clusters in our dataset. Unlike the chondrocytes, visualization of differentially expressed genes showed substantial overlap in the genes expressed by MSCs and proliferating MSCs, and by chondroprogenitors and multipotent stem cells (Fig. 6A). Chondroprogenitor markers did not show enrichment in other progenitor cells but did show enrichment in chondrocyte clusters, supporting that chondroprogenitors fall under a more chondrogenic lineage (Fig. 6B). Overlap in gene expression was observed between MSCs and proliferating MSCs but not in other clusters, supporting that they are closely related cell types (Fig. 6C, Fig. 6D). Multipotent stem cell markers were more uniquely expressed within that cluster but did show some enrichment in degenerated chondroprogenitors and degenerated NP cells (Fig. 6E).

**Fig. 7:**
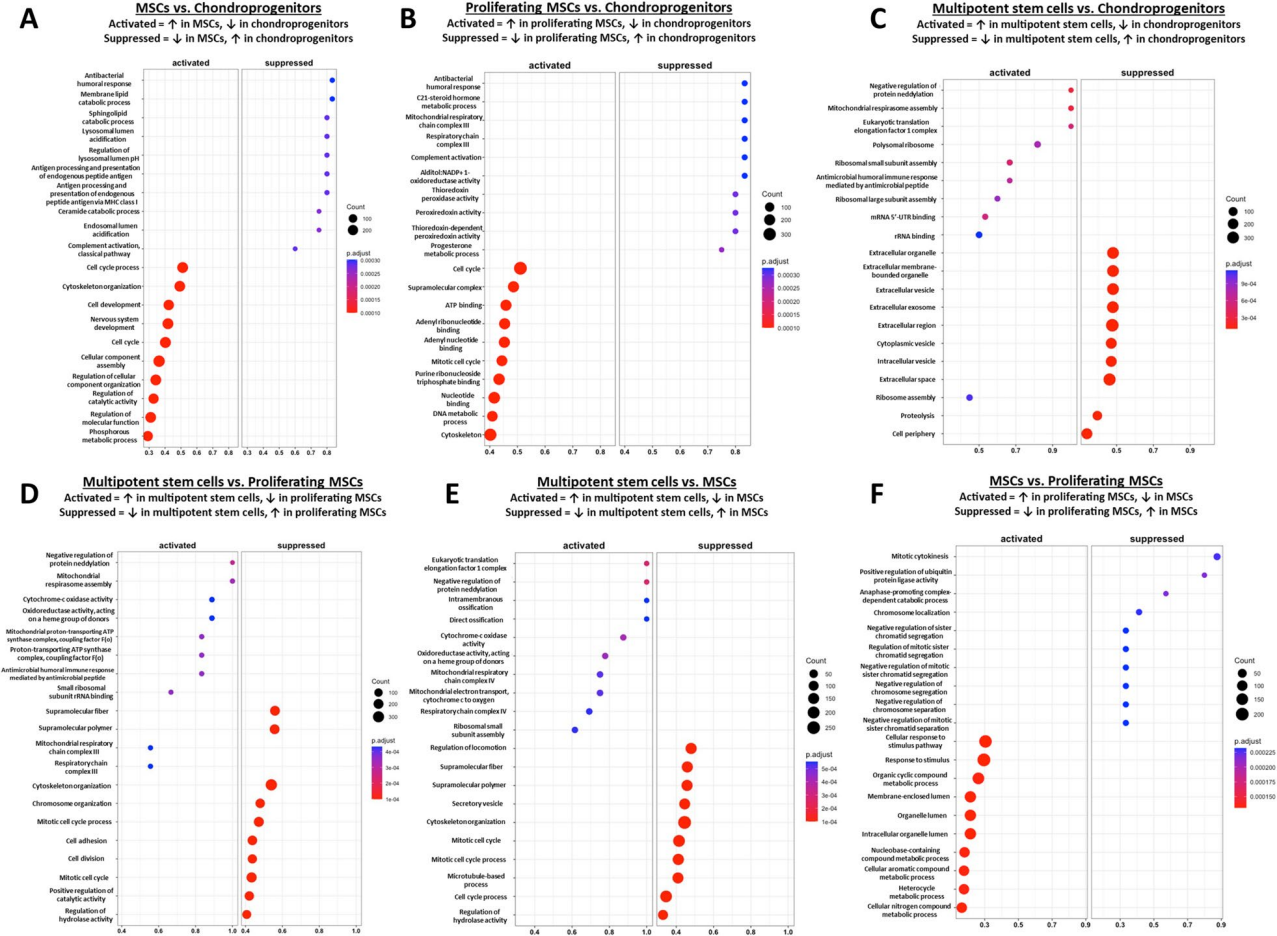
Gene ontology of biological processes comparing different progenitor cell clusters. **A** MSCs vs. chondroprogenitors. **B** Proliferating MSCs vs. chondroprogenitors. **C** Multipotent stem cells vs. chondroprogenitors. **D** Multipotent stem cells vs. proliferating MSCs. **E** Multipotent stem cells vs. MSCs. **F** MSCs vs. proliferating MSCs. For each figure, a description of “Activated” and “Suppressed” is provided

Chondroprogenitor cells were enriched in markers for maintenance of tissue ECM (*DCN*, *TIMP1*), regulators of oxidative stress (*SOD2*, *SOD3*, *IGFBP6*), heat shock proteins (*CLU*, *CRYAB*), and regulators of stemness and proliferation (*SPON2*, *SAT1*) (Fig. 6A). *CLU*, *CRYAB*, and *S100A1* are also upregulated during chondrogenesis [91–93] and *SAT1* may inhibit inflammation and hypertrophic differentiation of chondrocytes via depletion of polyamines [94]. Furthermore, *TIMP1* has an additional antiapoptotic role in various cell types [95]. Taken together, it appeared that chondroprogenitor cells in our dataset regulate tissue homeostasis and cellular stress. GO functional enrichment showed enrichment of lysosomal, endosomal, exosomal, mitochondrial, and immune response pathways (Fig. 7A, Fig. 7B, Fig. 7C). The endosome pathway interacts with lysosome and exosome functions, and the enrichment of lysosomes may suggest an increase in autophagy, which is important for cell survival in the IVD and cartilage [96].

Both MSCs and proliferating MSCs were enriched in pathways relating to cell division, but they may be at different stages of the cell cycle (Fig. 7A, Fig. 7B, Fig. 7D, Fig. 7E). Relative to proliferating MSCs, “normal” MSCs were enriched in pathways such as mitotic cytokinesis, anaphase-promoting complex-dependent catabolic process, chromosome localization, and various pathways related to chromatid/chromosome segregation and separation (Fig. 7F). They upregulated markers associated with microtubule dynamics (*STMN1*, *TUBA1B*, *PRC1*, *TPX2*), mitotic cyclin regulation (*CCNB1*, *CDKN3*), chromosome and chromatid organization (*CENPF*, *TOP2A*), and different phases of mitosis (*PRC1*, *STMN1*, *UBE2S*) (Fig. 6D). They also upregulated many genes associated with the different phases of mitosis (Fig. S3, Fig. S4, Fig. S5). Taken together, this may suggest that MSCs are in the process of entering or existing mitosis. Meanwhile, proliferating MSCs were more enriched in pathways such as organic cyclic compound metabolic process, nucleobase-containing compound metabolic process, heterocycle metabolic process, and cellular nitrogen compound metabolic process relative to “normal” MSCs (Fig. 7F); this may suggest an enrichment of nucleotide metabolism, as nucleotides consist of a heterocyclic nitrogenous base, an organic pentose molecule, and a phosphate group. They also upregulated various markers associated with DNA binding, replication, and repair (*CLSPN*, *HELLS*, *ATAD2*, *DNMT*, *RRM1*, *DEK*, *NSD2*) (Fig. 6C), which are important processes during the DNA synthesis phase (S phase) of the cell cycle [97]; there was a cluster-specific enrichment of G1/S phase markers in proliferating MSCs as well (Fig. S6, Fig. S7). Taken together, this may suggest that proliferating MSCs are more active in the S phase of the cell cycle.

Multipotent stem cells were enriched in numerous ribosomal and mitochondrial pathways (Fig. 7C–E) and upregulated numerous ribosomal markers (*RPS5*, *RPL32*, *RPL35A*, *RPS15A*, *RPL12*, *RPL29*, *RPS23*, *RPL28*, *RPS12*) (Fig. 6E). They may also be involved in further protein processing, as they upregulate *SERF2*, a marker that promotes protein aggregation, and *OST4*, which catalyzes the process of co-translational N-glycosylation of proteins to promote proper protein folding and transport [98, 99]. Interestingly, cells in this cluster were also enriched in pathways related to ossification, suggesting a possible role in regulating CEP calcification.

#### Progenitor cells upregulate genes in bulk RNA sequencing dataset relative to other cell types

Our bulk RNA sequencing results showed that non-degenerated and degenerated CEP cells have different gene expression profiles, though this analysis constitutes the average makeup of the CEP; however, CEP cells are a heterogeneous cell population that all contribute to the overall makeup of those bulk samples. To estimate how the identified subpopulations contribute to overall CEP cell phenotype, we studied their expression of the significantly D.E. genes identified in our bulk RNA sequencing analysis.

**Fig. 8:**
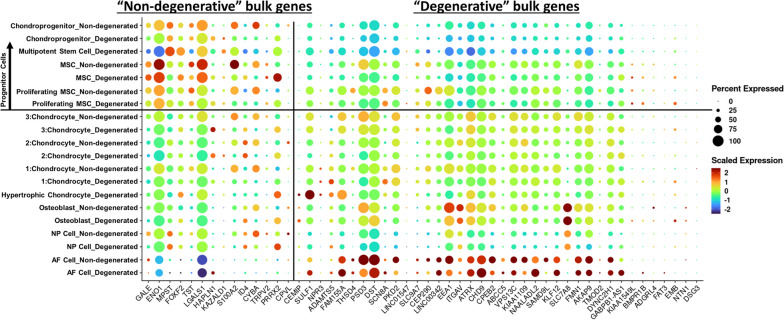
Dot plot of significantly D.E. markers with |F.C.| ≥ 1.5 identified in bulk RNA-Seq analysis. Vertical line separates the markers on the *x*-axis between “non-degenerated” bulk genes (left, F.C. < − 1.5) and “degenerative” bulk genes (right, F.C. > + 1.5). Horizontal line separates the clusters on the *y*-axis between progenitor cells (above) and more differentiated cells (below). D.E., differentially expressed; F.C., fold change

Cluster-by-cluster expression of genes upregulated in non-degenerated or degenerated bulk CEP samples were quantified (Table S7) and visualized using dot plots (Fig. 8). We first examined the expression of anabolic (*GALE*, *HAPLN1*) and catabolic (*ADAMTS5*, *CEMIP*) markers observed in our bulk RNA sequencing results. *GALE* was enriched in non-degenerated NP cells relative to degenerated NP cells but showed no notable sample-dependent enrichment in the remaining clusters. *HAPLN1*, however, was upregulated in degenerated chondrocyte 2, chondrocyte 3, proliferating MSC, and chondroprogenitor clusters relative to non-degenerated clusters; these results differed from our bulk RNA sequencing results. In contrast, *ADAMTS5* and *CEMIP* were enriched in hypertrophic chondrocyte and degenerated chondrocyte 1 clusters in cells of the hypertrophic chondrocyte and degenerated chondrocyte 1 clusters, which agreed with our bulk RNA sequencing results; furthermore, *ADAMTS5* was enriched in degenerated NP cells relative to non-degenerated NP cells and *CEMIP* was enriched in degenerated proliferating MSCs relative to non-degenerated cells. There were few notable differences in enrichment between non-degenerated and degenerated clusters for most other genes; *CPVL*, *CYBA*, and *S100A2* were enriched in most non-degenerated clusters relative to degenerated clusters, whereas *BMPR1B*, *EMB*, and *PRRX2* were enriched in most degenerated clusters relative to non-degenerated clusters. There was little notable enrichment or downregulation of “non-degenerated” or “degenerative” genes in all chondrocyte clusters. “Degenerative” bulk genes appeared to be downregulated in NP cells but somewhat enriched in osteoblasts relative to “non-degenerated” bulk genes. AF cells, despite accounting for a minor percentage of the cellular composition of our samples, greatly upregulated most “degenerative” bulk genes and downregulated most “non-degenerated” bulk genes.

Notably when grouping all progenitor cell clusters together (chondroprogenitors, multipotent stem cells, MSCs, and proliferating MSCs), most “non-degenerated” bulk genes were upregulated and most “degenerative” genes were downregulated. This is notable as mesenchymal stem cells can differentiate and secrete factors with therapeutic effects to augment regeneration of degenerated cartilage and IVD cells [100–103]. Additionally, a progenitor cell population was previously identified in CEP tissue and may exert pro-chondrogenic and anti-inflammatory effects on other disc cells [104–106]. Taken together, this may suggest that a non-degenerated CEP cell phenotype is driven primarily by progenitor cell subpopulations within the tissue.

#### Degenerated cells were enriched in genes associated with mitochondrial and ribosomal function

**Fig. 9:**
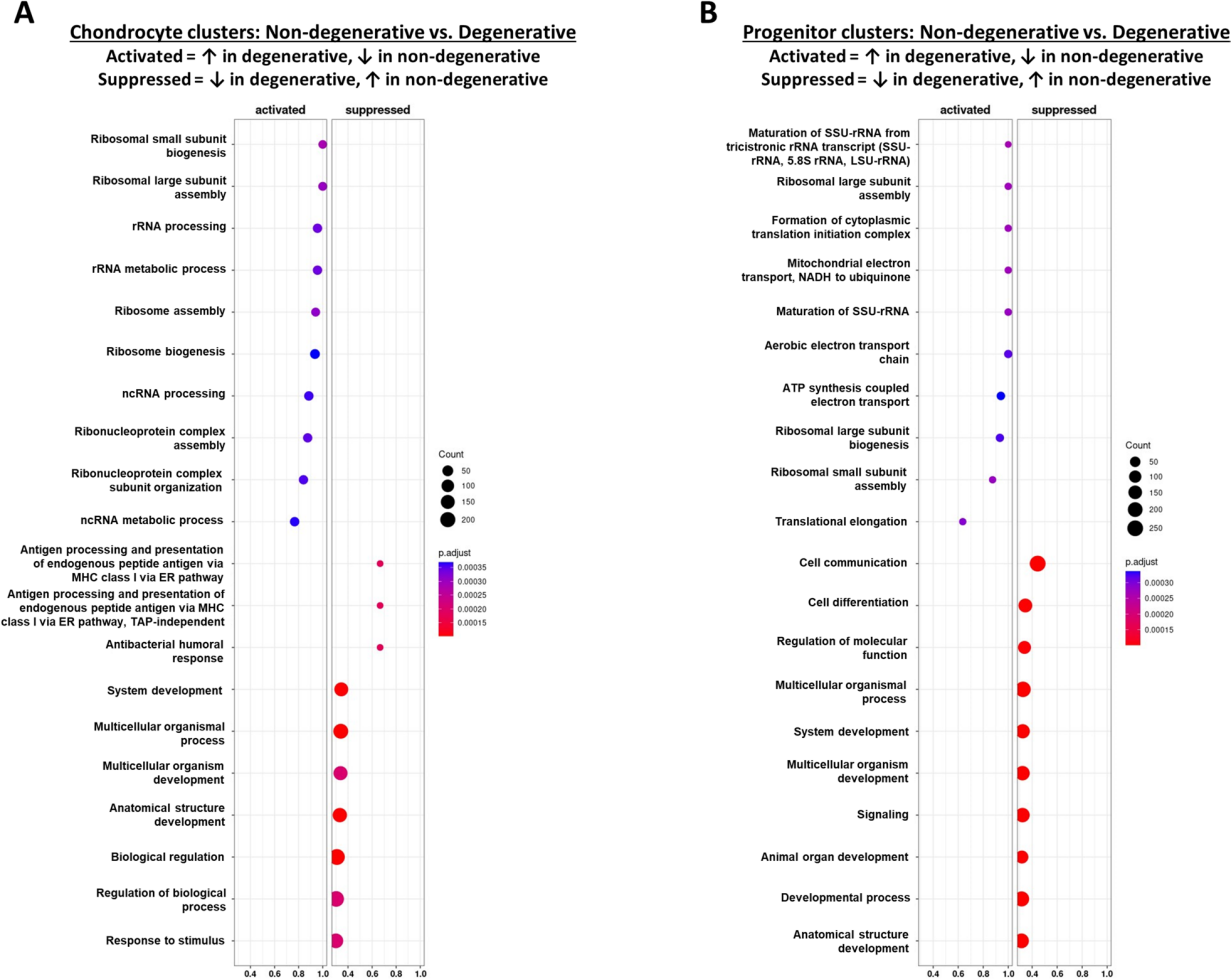
Gene ontology of biological processes comparing non-degenerated and degenerated cell clusters. **A** Non-degenerated chondrocytes 1, 2, and 3 (pooled) vs. degenerated chondrocytes 1, 2, and 3 (pooled). **B** Non-degenerated chondroprogenitors, MSCs, and proliferating MSCs (pooled) vs. degenerated chondroprogenitors, MSCs, and proliferating MSCs (pooled). For each panel, a description of “Activated” and “Suppressed” is provided

After characterizing the heterogeneity of non-degenerated and degenerated CEP cells, we finally studied the effects of degeneration on the different cell subtypes identified in this analysis. GO functional enrichment analysis showed that degenerated chondrocytes and degenerated progenitor cells were enriched in markers for ribosomal, translational, and oxidative phosphorylation-related pathways, whereas non-degenerated clusters were enriched for general cell-cell signaling, communication, and developmental pathways (Fig. 9A, Fig. 9B). Functional enrichment values and gene lists for these pathways are provided in Table S8 (chondrocyte pathways) and Table S9 (progenitor cell pathways). Comparisons between non-degenerated and degenerated cells for individual chondrocyte or progenitor cell clusters showed similar results (Fig. S8-S13). Low ribosomal and mitochondrial RNA content in our quality control analysis suggest these results were not caused by enrichment of ribosomes and mitochondria in our samples (Fig. S1).

## Discussion

Though the CEP has important mechanical and transport functions to regulate IVD degeneration, few studies have characterized the phenotype and functions of the resident CEP cells. While CEP cells resemble articular chondrocytes, several studies have demonstrated tissue and gene-level differences between the two [34, 38]; thus, CEP cells must be more thoroughly characterized to distinguish them from other cartilaginous cell types. Numerous groups have recently used RNA sequencing to characterize the cellularity and degenerative changes within AF and NP tissues [107–111], and Gan et al. [112] has performed single-cell RNA sequencing to examine the cellular profile of CEP tissue from a single donor. To expand on the existing body of knowledge, we performed bulk- and single-cell RNA sequencing on human CEP cells from numerous non-degenerated and degenerated IVDs; the current study investigated broad transcriptomic differences between non-degenerated and degenerated CEP cells, identified novel genes and pathways of interest for future study, and highlighted the phenotypic and functional heterogeneity of CEP cells.

Our bulk RNA sequencing analysis identified numerous differentially expressed genes between non-degenerated and degenerated CEP cells. We identified several anabolic ECM markers (*HAPLN1*, *GALE*) that were upregulated in non-degenerated cells and degradative ECM markers (*ADAMTS5*, *CEMIP*) that were upregulated in degenerative cells, consistent with prior findings showing an imbalance of ECM marker expression during cartilage and IVD degeneration [27, 54, 55]. Interestingly, all but 2 genes in the current dataset (*ADAMTS5*, *SULF1*) were not previously studied in CEP cells, including those that had and had not been previously studied in other cartilaginous tissues (i.e., articular cartilage, growth plate cartilage, IVD). While genes enriched in non-degenerated and degenerated cells both encompassed a broad array of functions, there were disparities in the types of genes enriched in both groups. Many genes enriched in non-degenerated CEP cells were associated with anabolic, metabolic, development, and cell cycling functions (*FOXF2*, *PRRX2*, *S100A2*, *ID4*, *ENO1*, *TST*). *FOXF2* and *PRRX2* are important factors for craniofacial development, and *S100A2* is expressed by chondrocyte-like cells and localizes to sites of calcifying cartilage, suggesting their role in skeletogenesis [111, 113–116]. *ID4* also regulates cell proliferation and differentiation in many developmental processes and is upregulated during chondrogenic differentiation [117, 118]. *ENO1* is a major metabolic enzyme that catalyzes the penultimate step of glycolysis and is upregulated by stimulation by the chondrogenic growth factor *CCN2* [119]; notably, glycolysis is the primary method of energy production in various cartilaginous tissues due to their hypoxic environments [120, 121]. *TST*, also known as rhodanese, is another important metabolic enzyme that localizes to the mitochondria and may regulate its function by maintaining redox homeostasis [122]; as mitochondrial dysfunction and reactive oxygen species buildup is observed in osteoarthritic cartilage and aged disc cells [123, 124], this may point to a critical role for *TST* in regulating metabolism in non-degenerated cells. Several of these genes also have pro-chondrogenic and chondro-protective roles (*GALE*, *HAPLN1*, *TRPV2*, *MPST*). *GALE* and *HAPLN1* are associated with synthesis and stabilization of proteoglycans within cartilaginous ECM [51, 52]. *TRPV2*, a mechanosensitive Ca^2+^ channel, and *MPST*, a sulfurtransferase that generates the gasotransmitter H_2_S, are protective against cartilage degradation and calcification in in vivo models of surgically induced osteoarthritis [56, 57]. Interestingly, many of these genes were also previously studied in other cartilaginous tissues.

Degenerated cells were enriched in genes that may regulate chondrocyte hypertrophy and calcification during endochondral ossification, including *BMPR1B*, *FMN1*, *NPR3*, and *CEMIP*; this is significant as calcification is a major feature of CEP degeneration that decreases tissue permeability and is proposed to occur via endochondral ossification [31, 67]. *BMPR1B* is a receptor for the chondrogenic growth factor GDF5 and can inhibit hypertrophic differentiation of chondrocytes [125]. *FMN1* regulates actin polymerization and regulates chondrocyte hypertrophy and migration during endochondral ossification [126]. *NPR3* is a decoy receptor for C-type natriuretic peptide, which is necessary for proper long bone growth [127]; *NPR3* deficiency results in elongated bones [127], suggesting a role in regulating endochondral ossification. *CEMIP* is a degradative enzyme that degrades hyaluronan to facilitate osteoclast and blood vessel invasion into hypertrophic cartilage and is also upregulated in osteoarthritic cartilage [53, 128].

Other genes upregulated in degenerated CEP cells and potentially associated with disease progression and pain were *ADAMTS5*, *NTN1*, *ITGAV*, and *THSD4*. *ADAMTS5* is a major enzyme that degrades the major structural proteoglycan aggrecan and is upregulated in degenerated CEP and other cartilaginous tissues [28]. *NTN1* promotes axon guidance in neurons and has been implicated in the ingrowth of sensory neurons in in vivo models of experimental disc degeneration and osteoarthritis [129, 130] and may also promote angiogenesis [131]. Interestingly, nerve ingrowth into the painful disc is usually localized within proteoglycan-depleted regions of tissue [10], highlighting the potential interplay between *ADAMTS5* and *NTN1*. *ITGAV* is a cell adhesion protein that is expressed by chondrocytes and NP cells [132, 133]. Interestingly, this particular integrin regulates mechanically regulated TGFβ signaling [134] and is upregulated in cartilage and the spine in experimental osteoarthritis and disc degeneration driven by excessive TGFβ signaling [135, 136]. Meanwhile, *THSD4* promotes the assembly of microfibrils, which can bind to latent TGFβ binding proteins to regulate the availability of active TGFβ [137, 138]. These results highlight how signaling pathways may become altered in CEP cells in association with degeneration and pain.

Many remaining genes enriched in degenerated CEP cells had not been studied previously in cartilaginous tissues and had signaling, transcriptional and translational regulation, and cytoskeleton and matrix regulation functions. Several of these genes (*CEP290*, *DYNC2H1*, *PKD2*) commonly localize to the primary cilia and are necessary for proper ciliogenesis, intraciliary transport, and ciliary mechanotransduction, indicating a possible significance in signal transduction [139–141]. Notably, there appeared to be enrichment of genes associated with endosomal and protein processing pathways (*SAMD9L*, *PSD3*, *EEA1*, *VPS13C*, *KIAA1109*, *SLC9A7*). Endosomes are important for general cell metabolism by functioning as sorting and transport centers for endocytosed and intracellular cargo and preparing it for subsequent processing, degradation, or exosomal re-export [142]. The endosomal pathway is also linked to autophagy, a process of recycling intracellular cargo to promote cell survival and is the common focus in various mechanistic studies of CEP degeneration [105, 143–146]. Our results appear to corroborate the possible significance of this pathway and highlight that alterations in intracellular signaling and metabolism might be prevalent in degenerated CEP cells.

Interestingly, our bulk RNA sequencing analysis found an enrichment of several cytoskeletal and membrane transport markers with neuron-specific enrichment (*SCN8A*, *FAM155A*, *KIAA1109*, *ABCC5*, *TMOD2*) in our degenerated bulk samples. However, we did not identify any neuronal cells despite screening for them in our single-cell analysis. Neural ingrowth into the CEP has been reported in painful IVDs and was found to occur primarily through degenerated, proteoglycan-depleted tissue [10, 13]. Enrichment of these markers in our samples suggests that those markers are naturally expressed by CEP cells, highlighting the use of RNA sequencing to discover novel genes in CEP cells and may suggest the importance of ion channels and cytoskeletal proteins in CEP cell function.

The current single-cell analysis identified various chondrocyte clusters. These were the most plentiful cell types in our samples and each appeared to have distinct functions, including signal regulation, biosynthesis, and pro-chondrogenic or pre-hypertrophic functions. This highlights the cellular heterogeneity within CEP cell samples and presents multiple cellular targets that could contribute to CEP health and degeneration, though it remains to be determined the significance of these different clusters. Notably, Gan et al. [112] also found that the predominant cell types in one CEP sample were 3 primary chondrocyte-like clusters. Among these were “regulatory chondrocytes” that upregulated growth factors and chondrogenic pathway regulators, “homeostatic chondrocytes” that upregulated ECM proteins and circadian rhythm markers, and “effector chondrocytes” that had a more hypertrophic character; this was similar to our findings and supports the presence of these cell subtypes in CEP tissue.

We also identified a chondroprogenitor cluster, two subtypes of mesenchymal stem cells, and another multipotent stem cell cluster. Resident progenitor cells similar to mesenchymal stem cells have been previously identified in articular cartilage [147], the NP [148], and the CEP [104] and are believed to contribute to tissue homeostasis and regeneration; these progenitors have increased chondrogenic potential compared to those isolated from other common stem cell sources and actively proliferate. Mesenchymal stem cells also secrete therapeutic factors that can mitigate tissue injury and inflammation in osteoarthritis and IVD degeneration [100–103, 105]. In our dataset, our mesenchymal stem cell clusters were actively proliferative whereas our chondroprogenitors upregulated markers associated with ECM maintenance and regulation of oxidative stress and protein aggregation; these results may suggest that progenitor cells responsible for proliferating and secreting therapeutic factors belong to different subpopulations and therefore have different roles in maintaining tissue homeostasis.

Interestingly, our chondroprogenitors were enriched in pathways such as extracellular exosome and those associated with endosomes. Exosomes are small membrane-enclosed structures that can modulate cell activity via the transfer of various biological cargo and are secreted from multivesicular bodies derived from late endosomes [149, 150]. Stem cell-derived EVs have been studied for their therapeutic effects in various diseases, including arthritis and disc degeneration [100–103]. Notably, exosomes have been isolated from rat CEP cells and were demonstrated to have anti-inflammatory and pro-chondrogenic effects on other disc cells [105, 106], suggesting a possible role for EV-mediated functions for CEP-derived progenitor cells. The enrichment of endosomal-related pathways may also point toward an increase in lysosomal and autophagy-related pathways, thereby contributing to cell survival [96].

Multipotent stem cells were only present in our degenerated sample and were enriched in ribosomal, protein translation, and mitochondrial function pathways. Interestingly, all clusters from our degenerated CEP sample were also enriched in similar types of pathways. Ribosomes are organelles that translate proteins from mRNA transcripts and can therefore regulate protein expression, and their role in regulating cartilaginous disease is gaining new attention [151]. Osteoarthritic and aged chondrocytes exhibit altered ribosome biogenesis and processing, which can regulate their response to oxidative stress and other stimuli [151–153]. Whereas chondrocytes typically translate proteins via a 5′ cap-dependent mechanism, chondrocytes exposed to inflammatory stimuli (i.e., cytokines, osteoarthritic synovial fluid) upregulate translation through an alternative IRES-dependent mechanism that is commonly activated in various stress conditions (i.e., endoplasmic reticulum stress, nutrient deprivation, inflammation, apoptosis, mitosis) to produce stress-related proteins [151, 154–157]. Currently, the association between proper protein translation dynamics and IVD and CEP degeneration is understudied. However, Yang et al. [158] found that several ribosomal proteins and protein translation pathways were differentially expressed between healthy and degenerated IVD cells. This, in coordination with our findings of enrichment of genes associated with protein translation pathways in degenerated CEP cell clusters, indicates ribosome function and protein translation dynamics may be novel factors regulating CEP degeneration.

Mitochondria generate ATP through oxidative phosphorylation and are therefore important for energy metabolism. Articular chondrocytes and NP cells exist in hypoxic environments and preferentially generate ATP via glycolysis [120, 121]. The NP directly underlying the CEP in canine discs has an oxygen tension ~15% that of blood oxygen tension [159], which may suggest the CEP is hypoxic as well and may prefer glycolytic pathways. Though we expanded both non-degenerated and degenerated CEP cells at 21% O_2_, only degenerated cells were enriched in pathways associated with mitochondrial function and oxidative phosphorylation. Altered mitochondrial function is associated with various diseases, including degeneration of cartilaginous tissues [160, 161]. Some studies have reported that mitochondrial function is reduced in degenerated IVD cells and osteoarthritic cartilage, while upregulation of oxidative phosphorylation has a chondroprotective effect in articular chondrocytes [123, 124]. In contrast, Cisewski et al. reported elevated oxygen consumption rates indicative of increased oxidative phosphorylation in degenerated IVD and CEP cells relative to healthy cells [162]. These reports suggest there could be multiple modes of mitochondrial dysfunction that are associated with IVD degeneration or it may serve as a compensatory mechanism to alleviate degenerative changes.

These results provide a new and substantial body of information expanding our current knowledge of CEP cells, but their impact may be affected by several limitations. We utilized cultured CEP cells instead of freshly isolated cells in these experiments to increase our cell count for RNA sequencing experiments due to low yield of viable cells from freshly isolated human CEP tissue. The results here could be used as a reference for the many experiments that use cultured CEP cells, as monolayer cell culture is a common model used to study CEP cells. However, cell expansion in monolayer could result in a loss of the native CEP cell phenotype through dedifferentiation [163] and may lead to a loss of less adherent cell subpopulations. We attempted to minimize the effects of cell culture by using cells that have been minimally passaged. Though CEP cell phenotype post-expansion was not evaluated prior to RNA sequencing, CEP cells passaged once after isolation continue to express chondrogenic markers *ACAN* and *COL2A1* at similar or elevated levels to articular chondrocytes and NP cells [34], suggesting some maintenance of a native phenotype; furthermore, non-degenerated and degenerated disc cells first expanded in monolayer still have inherent differences post-expansion, suggesting similar differences may be detectable in our dataset [12, 164]. The use of human cells increases the clinical utility of the current work, but it may also increase the variability of the results due to the different demographics of human subjects the samples were sourced from. While this may make it more difficult to identify all differences between non-degenerated and degenerated samples, the future expansion of the current dataset to account for all demographic differences (i.e., sex, age, race, lifestyle) can highlight new findings. We used the macroscopic Thompson scale to grade IVDs and classify CEP cells as “non-degenerated” or “degenerated,” though CEP structure has only a minor influence on scoring and may therefore not reflect the actual degenerative status of our samples [165]. Furthermore, each sample included cells from both the cranial and caudal CEP, which may have different degeneration patterns. Finally, while we did not validate our sequencing results using other quantitative measures such as qRT-PCR or protein assays, the goal of the current work was to explore CEP cell phenotype, highlight novel markers, and identify possible markers that are differentially expressed in non-degenerated and degenerated CEP cells; future experiments will be performed that validate the findings of this sequencing data.

## Conclusions

We identified numerous differentially expressed genes between non-degenerated and degenerated CEP cells using bulk RNA sequencing and highlighted many novel genes not previously reported on in the CEP or other cartilaginous tissues. We reviewed the genes in our dataset and pinpointed several pathways of interest, including transcriptional regulation, translational regulation, intracellular transport, and mitochondrial pathways, that may be worth investigating in future experiments. We also characterized the cellular profile of non-degenerated and degenerated CEP cell samples using single-cell RNA sequencing and found numerous subpopulations of chondrocyte-like cells and progenitor cells. Notably, we found that progenitor cells had higher expression of many genes upregulated in non-degenerated CEP cells and lower expression of many genes upregulated by degenerated CEP cells. This work addresses a critical gap in clarifying the phenotype of CEP cells and could act as a stepping-stone to identify novel markers that regulate CEP degeneration, as well as identify novel CEP-specific markers for the development of preclinical models or CEP-targeted therapeutics. This will significantly impact how we understand the role of the CEP in regulating IVD joint degeneration and discogenic back pain.

### Supplementary Information

**Additional file 1: Table S1.** Number of publications, search terms, and PubMed IDs for all identified papers for all genes in each tissue of interest.

**Additional file 2: Table S2.** Fold change and adjusted p-values for all significantly D.E. genes. **Table S3.** General breakdown of genes with prior publications in cartilaginous cells (*N*=31 total genes, *q*<0.05). **Table S4.** General breakdown of genes with no prior publications in cartilaginous cells (*N*=45 total genes, *q*<0.05). **Table S5.** Screening of cell types and corresponding markers possibly isolated in CEP samples. **Table S6.** Cell types and corresponding markers identified in current single-cell RNA-Sequencing analysis.

**Additional file 3: Figure S1.** Pre-filtering quality control. Violin plots showing RNA unique features, total RNA count, mitochondrial RNA content, ribosomal RNA content, hemoglobin RNA content, and platelet RNA content for all cells are shown.

**Additional file 4: Table S7.** Percentage expressed and scaled expressed of differentially expressed genes from bulk RNA sequencing analysis, by cluster. 

**Additional file 5: Table S8.** Functional enrichment values and gene lists for Gene Ontology pathways, in chondrocytes.

**Additional file 6: Table S9.** Functional enrichment values and gene lists for Gene Ontology pathways, in progenitor cells.

**Additional file 7: Figure S2.** Dot plot of top 3 markers of each cluster identified in single-cell RNA-Sequencing analysis.

**Additional file 8: Figure S3.** Dot plots of markers associated with different phases of mitosis. Markers were selected from the following gene sets from https://maayanlab.cloud/Harmonizome/: 1) Prophase (GeneRIF Biological Term Annotations), 2) Prometaphase (GeneRIF Biological Term Annotations), and 3) Metaphase (GeneRIF Biological Term Annotations).

**Additional file 9: Figure S4.** Dot plots of markers associated with different phases of mitosis. Markers were selected from the following genes sets from https://maayanlab.cloud/Harmonizome/: 1) Anaphase (GeneRIF Biological Term Annotations), 2) Telophase (GeneRIF Biological Term Annotations), and 3) Cytokinesis (GO Biological Process Annotations).

**Additional file 10: Figure S5.** Module score uMAP of mitosis-related genes from Fig. S3 and Fig. S4.

**Additional file 11: Figure S6.** Dot plot of markers associated with G1/S phase of cell cycle. Markers were selected from gene sets from https://maayanlab.cloud/Harmonizome/.

**Additional file 12: Figure S7.** Module score uMAP of G1/S transition genes from Fig. S6.

**Additional file 13: Figure S8.** Gene ontology comparing Non-degenerated and Degenerated Chondrocyte 1. Red boxes indicate pathways associated with ribosomes, protein translation, and mitochondrial function. “Activated” pathways are enriched in the degenerated sample and “Suppressed” pathways are enriched in the non-degenerated sample.

**Additional file 14: Figure S9.** Gene ontology comparing Non-degenerated and Degenerated Chondrocyte 2. Red boxes indicate pathways associated with ribosomes, protein translation, and mitochondrial function. “Activated” pathways are enriched in the degenerated sample and “Suppressed” pathways are enriched in the non-degenerated sample.

**Additional file 15: Figure S10.** Gene ontology comparing Non-degenerated and Degenerated Chondrocyte 3. Red boxes indicate pathways associated with ribosomes, protein translation, and mitochondrial function. “Activated” pathways are enriched in the degenerated sample and “Suppressed” pathways are enriched in the non-degenerated sample.

**Additional file 16: Figure S11.** Gene ontology comparing Non-degenerated and Degenerated Chondroprogenitors. Red boxes indicate pathways associated with ribosomes, protein translation, and mitochondrial function. “Activated” pathways are enriched in the degenerated sample and “Suppressed” pathways are enriched in the non-degenerated sample.

**Additional file 17: Figure S12.** Gene ontology comparing Non-degenerated and Degenerated MSCs. Red boxes indicate pathways associated with ribosomes, protein translation, and mitochondrial function. “Activated” pathways are enriched in the degenerated sample and “Suppressed” pathways are enriched in the non-degenerated sample.

**Additional file 18: Figure S13.** Gene ontology comparing Non-degenerated and Degenerated Proliferating MSCs. Red boxes indicate pathways associated with ribosomes, protein translation, and mitochondrial function. “Activated” pathways are enriched in the degenerated sample and “Suppressed” pathways are enriched in the non-degenerated sample.

## Data Availability

All figures and tables referenced in this study are available within this published article or available as supplementary files. Raw bulk (GSE242040) and single-cell RNA sequencing data (GSE242443) are publicly available in the GEO database.

## Abbreviations

AF: Annulus fibrosus
BSA: Bovine serum albumin
CEP: Cartilage endplate
D.E.: Differentially expressed
DMEM: Dulbecco’s modified Eagle medium
ECM: Extracellular matrix
FBS: Fetal bovine serum
GEM: Gel bead-in-emulsion
GO: Gene Ontology
IVD: Intervertebral disc
NP: Nucleus pulposus
PCR: Polymerase chain reaction
RIN: RNA integrity number
