# Supplementary material for: Transcriptional profiling of human cartilage endplate cells identifies novel genes and cell clusters underlying degenerated and non-degenerated phenotypes
Source: Arthritis Res Ther. 2024 Jan 3;26:12. doi: 10.1186/s13075-023-03220-6 (PMC10763221; doi:10.1186/s13075-023-03220-6)
Supplement: Supplementary file 2 — Additional file 2: Table S2. Fold change and adjusted p-values for all significantly D.E. genes. Table S3. General breakdown of genes with prior publications in cartilaginous cells (N=31 total genes, q<0.05). Table S4. General breakdown of genes with no prior publications in cartilaginous cells (N=45 total genes, q<0.05). Table S5. Screening of cell types and corresponding markers possibly isolated in CEP samples. Table S6. Cell types and corresponding markers identified in current single-cell RNA-Sequencing analysis. [file 13075_2023_3220_MOESM2_ESM.docx]

| **Table S2: Fold change and adjusted p-values for all significantly D.E. genes** | | | | |
| --- | --- | --- | --- | --- |
| **Gene name** | **Gene status** | **log2FC** | **Fold change** | **Adj. p-value** |
| AL450405.1 | Non-degenerated, \|Fold Change\|≥1.5 | -2.0506 | 0.2414 | 0.0368 |
| CPVL | Non-degenerated, \|Fold Change\|≥1.5 | -1.9804 | 0.2534 | 0.0004 |
| PRRX2 | Non-degenerated, \|Fold Change\|≥1.5 | -1.6129 | 0.3269 | 0.0461 |
| TRPV2 | Non-degenerated, \|Fold Change\|≥1.5 | -1.5958 | 0.3308 | 0.0004 |
| CYBA | Non-degenerated, \|Fold Change\|≥1.5 | -1.2239 | 0.4281 | 0.0001 |
| ID4 | Non-degenerated, \|Fold Change\|≥1.5 | -1.1558 | 0.4488 | 0.0375 |
| S100A2 | Non-degenerated, \|Fold Change\|≥1.5 | -1.0920 | 0.4691 | 0.0001 |
| KAZALD1 | Non-degenerated, \|Fold Change\|≥1.5 | -0.9624 | 0.5132 | 0.0001 |
| HAPLN1 | Non-degenerated, \|Fold Change\|≥1.5 | -0.8936 | 0.5383 | 0.0072 |
| LGALS1 | Non-degenerated, \|Fold Change\|≥1.5 | -0.8106 | 0.5701 | 0.0348 |
| TST | Non-degenerated, \|Fold Change\|≥1.5 | -0.7957 | 0.5761 | 0.0272 |
| FOXF2 | Non-degenerated, \|Fold Change\|≥1.5 | -0.7860 | 0.5799 | 0.0367 |
| MPST | Non-degenerated, \|Fold Change\|≥1.5 | -0.7079 | 0.6122 | 0.0072 |
| ENO1 | Non-degenerated, \|Fold Change\|≥1.5 | -0.6914 | 0.6193 | 0.0111 |
| GALE | Non-degenerated, \|Fold Change\|≥1.5 | -0.6013 | 0.6592 | 0.0175 |
| MANF | Non-degenerated, \|Fold Change\|<1.5 | -0.5745 | 0.6715 | 0.0457 |
| DBI | Non-degenerated, \|Fold Change\|<1.5 | -0.5169 | 0.6989 | 0.0368 |
| SLC2A4RG | Non-degenerated, \|Fold Change\|<1.5 | -0.5139 | 0.7003 | 0.0217 |
| CAVIN3 | Non-degenerated, \|Fold Change\|<1.5 | -0.4860 | 0.7140 | 0.0240 |
| S100A10 | Non-degenerated, \|Fold Change\|<1.5 | -0.4625 | 0.7257 | 0.0266 |
| SCCPDH | Non-degenerated, \|Fold Change\|<1.5 | -0.4489 | 0.7326 | 0.0368 |
| EIF6 | Non-degenerated, \|Fold Change\|<1.5 | -0.4463 | 0.7339 | 0.0368 |
| EMD | Non-degenerated, \|Fold Change\|<1.5 | -0.3957 | 0.7601 | 0.0261 |
| SYNJ2 | Degenerated, \|Fold Change\|<1.5 | 0.3934 | 1.3135 | 0.0241 |
| MYOF | Degenerated, \|Fold Change\|<1.5 | 0.4273 | 1.3447 | 0.0368 |
| TTC37 | Degenerated, \|Fold Change\|<1.5 | 0.4364 | 1.3532 | 0.0348 |
| CLIP1 | Degenerated, \|Fold Change\|<1.5 | 0.4379 | 1.3546 | 0.0055 |
| MT-ND5 | Degenerated, \|Fold Change\|<1.5 | 0.4515 | 1.3675 | 0.0368 |
| CAPRIN2 | Degenerated, \|Fold Change\|<1.5 | 0.4661 | 1.3814 | 0.0368 |
| EIF4G3 | Degenerated, \|Fold Change\|<1.5 | 0.4689 | 1.3841 | 0.0461 |
| OSBPL8 | Degenerated, \|Fold Change\|<1.5 | 0.4716 | 1.3866 | 0.0065 |
| MIA3 | Degenerated, \|Fold Change\|<1.5 | 0.4874 | 1.4019 | 0.0461 |
| DMXL1 | Degenerated, \|Fold Change\|<1.5 | 0.5017 | 1.4159 | 0.0368 |
| ARHGAP31 | Degenerated, \|Fold Change\|<1.5 | 0.5046 | 1.4187 | 0.0348 |
| PIK3C2A | Degenerated, \|Fold Change\|<1.5 | 0.5074 | 1.4215 | 0.0348 |
| AGL | Degenerated, \|Fold Change\|<1.5 | 0.5422 | 1.4562 | 0.0300 |
| SOS1 | Degenerated, \|Fold Change\|<1.5 | 0.5650 | 1.4794 | 0.0001 |
| GCC2 | Degenerated, \|Fold Change\|<1.5 | 0.5678 | 1.4823 | 0.0241 |
| EEA1 | Degenerated, \|Fold Change\|≥1.5 | 0.6056 | 1.5216 | 0.0029 |
| ITGAV | Degenerated, \|Fold Change\|≥1.5 | 0.6299 | 1.5475 | 0.0184 |
| ATRX | Degenerated, \|Fold Change\|≥1.5 | 0.6501 | 1.5693 | 0.0072 |
| CHD9 | Degenerated, \|Fold Change\|≥1.5 | 0.6539 | 1.5734 | 0.0220 |
| CPEB2 | Degenerated, \|Fold Change\|≥1.5 | 0.6712 | 1.5924 | 0.0028 |
| ABCC5 | Degenerated, \|Fold Change\|≥1.5 | 0.6793 | 1.6013 | 0.0017 |
| VPS13C | Degenerated, \|Fold Change\|≥1.5 | 0.6905 | 1.6138 | 0.0345 |
| KIAA1109 | Degenerated, \|Fold Change\|≥1.5 | 0.7003 | 1.6249 | 0.0175 |
| NAALADL2 | Degenerated, \|Fold Change\|≥1.5 | 0.7088 | 1.6344 | 0.0329 |
| LINC00342 | Degenerated, \|Fold Change\|≥1.5 | 0.7341 | 1.6634 | 0.0353 |
| SAMD9L | Degenerated, \|Fold Change\|≥1.5 | 0.7368 | 1.6664 | 0.0368 |
| KLF12 | Degenerated, \|Fold Change\|≥1.5 | 0.7665 | 1.7011 | 0.0241 |
| CEP290 | Degenerated, \|Fold Change\|≥1.5 | 0.7961 | 1.7364 | 0.0002 |
| SLC7A8 | Degenerated, \|Fold Change\|≥1.5 | 0.8031 | 1.7449 | 0.0016 |
| SLC9A7 | Degenerated, \|Fold Change\|≥1.5 | 0.8134 | 1.7574 | 0.0034 |
| FMN1 | Degenerated, \|Fold Change\|≥1.5 | 0.8560 | 1.8100 | 0.0111 |
| AKAP9 | Degenerated, \|Fold Change\|≥1.5 | 0.9081 | 1.8766 | 0.0014 |
| LINC01547 | Degenerated, \|Fold Change\|≥1.5 | 0.9467 | 1.9275 | 0.0055 |
| TMOD2 | Degenerated, \|Fold Change\|≥1.5 | 0.9553 | 1.9390 | 0.0240 |
| DYNC2H1 | Degenerated, \|Fold Change\|≥1.5 | 0.9784 | 1.9703 | 0.0111 |
| PKD2 | Degenerated, \|Fold Change\|≥1.5 | 0.9845 | 1.9787 | 0.0021 |
| SCN8A | Degenerated, \|Fold Change\|≥1.5 | 0.9975 | 1.9966 | 0.0368 |
| DST | Degenerated, \|Fold Change\|≥1.5 | 1.0358 | 2.0503 | 0.0034 |
| PSD3 | Degenerated, \|Fold Change\|≥1.5 | 1.0543 | 2.0768 | 0.0368 |
| GABPB1-AS1 | Degenerated, \|Fold Change\|≥1.5 | 1.2054 | 2.3060 | 0.0028 |
| THSD4 | Degenerated, \|Fold Change\|≥1.5 | 1.4289 | 2.6925 | 0.0000 |
| FAM155A | Degenerated, \|Fold Change\|≥1.5 | 1.4301 | 2.6946 | 0.0348 |
| ADAMTS5 | Degenerated, \|Fold Change\|≥1.5 | 1.5316 | 2.8911 | 0.0005 |
| NPR3 | Degenerated, \|Fold Change\|≥1.5 | 1.5734 | 2.9761 | 0.0002 |
| SULF1 | Degenerated, \|Fold Change\|≥1.5 | 1.7358 | 3.3307 | 0.0240 |
| KIAA1549L | Degenerated, \|Fold Change\|≥1.5 | 1.7602 | 3.3875 | 0.0348 |
| BMPR1B | Degenerated, \|Fold Change\|≥1.5 | 1.9623 | 3.8968 | 0.0017 |
| ADGRL4 | Degenerated, \|Fold Change\|≥1.5 | 2.1169 | 4.3375 | 0.0375 |
| FAT3 | Degenerated, \|Fold Change\|≥1.5 | 2.4543 | 5.4805 | 0.0052 |
| EMB | Degenerated, \|Fold Change\|≥1.5 | 2.5799 | 5.9788 | 0.0406 |
| NTN1 | Degenerated, \|Fold Change\|≥1.5 | 2.8941 | 7.4340 | 0.0028 |
| CEMIP | Degenerated, \|Fold Change\|≥1.5 | 3.9166 | 15.1018 | 0.0000 |
| DSG3 | Degenerated, \|Fold Change\|≥1.5 | 4.4236 | 21.4606 | 0.0077 |

*Fold Change values are calculated as Degenerated/Non-degenerated. |Fold Change|=Magnitude of fold change.

| **Table S3: General breakdown of genes with prior publications in cartilaginous cells (N=31 total genes, q<0.05)** | |
| --- | --- |
| **Category** | **Genes** |
| Ligand/receptor binding, signaling, chemotaxis | **Non-degenerated**: ----  **Degenerated**: BMPR1B, NTN1, NPR3  **\|Fold Change\|<1.5**: ---- |
| Signal transduction (GPRC, 2^nd^ messengers, etc.) | **Non-degenerated**: ----  **Degenerated**: ----  **\|Fold Change\|<1.5**: SOS1, PIK3C2A |
| Intracellular and/or membrane transport | **Non-degenerated**: TRPV2  **Degenerated**: PKD2  **\|Fold Change\|<1.5**: MIA3 |
| Transcription, RNA-binding, and translation regulation | **Non-degenerated**: FOXF2  **Degenerated**: ATRX, CHD9  **\|Fold Change\|<1.5**: ---- |
| Metabolism, proliferation, differentiation, and cell cycle | **Non-degenerated**: ENO1, PRRX2, TST, MPST, GALE, S100A2, ID4  **Degenerated**: ----  **\|Fold Change\|<1.5**: MANF, S100A10, MYOF |
| Cytoskeleton, ECM, and cell/matrix adhesion | **Non-degenerated**: HAPLN1, LGALS1  **Degenerated**: ADAMTS5, CEMIP, ITGAV, FMN1, FAT3  **\|Fold Change\|<1.5**: ---- |
| Proteolysis, protein-binding, and protein modification activity | **Non-degenerated**: ----  **Degenerated**: SULF1  **\|Fold Change\|<1.5**: ---- |
| Immunogenic, inflammatory, and stress function | **Non-degenerated**: CPVL  **Degenerated**: ----  **\|Fold Change\|<1.5**: CAVIN3 |
| Unclear function | **Non-degenerated**: ----  **Degenerated**: ----  **\|Fold Change\|<1.5**: ---- |

*Fold Change values are calculated as Degenerated/Non-degenerated. |Fold Change|=Magnitude of fold change.

| **Table S4: General breakdown of genes with no prior publications in cartilaginous cells (N=45 total genes, q<0.05)** | |
| --- | --- |
| **Category** | **Genes** |
| Ligand/receptor binding, signaling, chemotaxis | **Non-degenerated**: KAZALD1  **Degenerated**: SAMD9L  **\|Fold Change\|<1.5**: DBI |
| Signal transduction (GPRC, 2^nd^ messengers, etc.) | **Non-degenerated**: ----  **Degenerated**: ADGRL4, PSD3  **\|Fold Change\|<1.5**: ARHGAP31 |
| Intracellular and/or membrane transport | **Non-degenerated**: ----  **Degenerated**: FAM155A, SCN8A, SLC7A8, SLC9A7, EEA1, VPS13C, DYNC2H1, KIAA1109, ABCC5, CEP290  **\|Fold Change\|<1.5**: SYNJ2, OSBPL8, GCC2 |
| Transcription, RNA-binding, and translation regulation | **Non-degenerated**: ----  **Degenerated**: KLF12, CPEB2, GABPB1-AS1, LINC01547, LINC00342  **\|Fold Change\|<1.5**: SLC2A4RG, EIF6, EIF4G3, CAPRIN2 |
| Metabolism, proliferation, differentiation, and cell cycle | **Non-degenerated**: ----  **Degenerated**: ----  **\|Fold Change\|<1.5**: MT-ND5, SCCPDH, AGL |
| Cytoskeleton, ECM, and cell/matrix adhesion | **Non-degenerated**: ----  **Degenerated**: DSG3, THSD4, DST, TMOD2, EMB, AKAP9  **\|Fold Change\|<1.5**: EMD, CLIP1 |
| Proteolysis, protein-binding, and protein modification activity | **Non-degenerated**: ----  **Degenerated**: ----  **\|Fold Change\|<1.5**: TTC37 |
| Immunogenic, inflammatory, and stress function | **Non-degenerated**: CYBA  **Degenerated**: ----  **\|Fold Change\|<1.5**: ---- |
| Unclear function | **Non-degenerated**: AL450405.1  **Degenerated**: KIAA1549L, NAALADL2  **\|Fold Change\|<1.5**: DMXL1 |

*Fold Change values are calculated as Degenerated/Non-degenerated. |Fold Change|=Magnitude of fold change.

| **Table S5: Screening of cell types and corresponding markers possibly isolated in CEP samples** | | |
| --- | --- | --- |
| **Cell Category** | **Cell type** | **Markers** |
| **Disc cells** | Chondrocyte | SOX5, SOX6, SOX9, COL2A1, ACAN, COMP, MIA, COL9A1, LECT1 |
|  | NP cell | PAX1, FOXF1, KRT8, KRT18, CA12, LGALS3, CD24 |
|  | Notochord | KRT8, KRT18, KRT19, SHH, TBXT, FOXA2, CHRD, NOG, CDH2 |
|  | AF cell | MKX, SCX, COL12A1, SFRP2, COL5A1, COL1A1, TNMD |
| **Possible contaminants** | Muscle | CNN1, ACTA2, TAGLN, CALD1, SMTN, MYLK |
|  | Endothelial cell | CDH5, LMO2, PECAM1, KDR, FLT1, VWF, CXCR4 |
|  | Adipose | LEP, HOXC8, HOXC9, ADIPOQ, SLC7A10, SLC27A1 |
|  | Neuronal | CGRP, UCHL1, NES |
| **Stromal and mesenchymal cells** | Mesenchymal stem cell | ITGB1, CD44, NT5E, THY1, ENG, VCAM1, MCAM, ALCAM, LEPR |
|  | Chondroprogenitor | TEK, GD2, PDGFRA, PDGFRB, PROCR, MCAM, SOX9, PRG4 |
|  | Fibroblast | FAP, S100A4, COL1A1, COL5A1, FBLN1, HSP47, THY1 |
|  | Osteoblast | RUNX2, SP7, COL1A1, ALPL, SPP1, SPARC, BGLAP, GJA1 |
|  | Osteocyte | DMP1, MEPE, PDPN, SOST, PHEX, CAPG, HYOU1, DSTN, FGF23 |
|  | Hypertrophic chondrocyte | SPP1, VEGFA, MEF2C, MMP13, COL10A1, MMP9, IHH |
| **Hematopoietic and Immune cells** | Macrophage | CD68, ITGAM, CD163, CD64, SPI1, CD14 |
|  | Mast cell | FCGR2B, CD33, KIT, ENPP3, FCER1A |
|  | Megakaryocyte | ITGA2B, GP9, GP1BA, ITGB3 |
|  | Hematopoietic stem cell | CD34, TEK, KIT, THY1, ITGA2B, SLAMF1, CD48, PTPRC, PROCR |

| **Table S6: Cell types and corresponding markers identified in current single-cell RNA-Sequencing analysis** | | |
| --- | --- | --- |
| **Cluster #** | **Annotation** | **Markers** |
| 1 | Chondroprogenitor cells | MCAM, NOTCH1, CD9, TGFB1, SOX9, TUBB, COL1A1, PDPN, TIMP1, GAPDH |
| 2 | 1:Chondrocyte | SOX5, SOX6, SOX9, COL2A1, ACAN, COMP, COL9A1, COL11A1, MMP3, COL1A1 |
| 3 | 2:Chondrocyte | SOX5, SOX6, SOX9, COL2A1, ACAN, COMP, COL9A1, COL11A1, MMP3, COL1A1 |
| 4 | 3:Chondrocyte | SOX5, SOX6, SOX9, COL2A1, ACAN, COMP, COL9A1, COL11A1, MMP3, COL1A1 |
| 5 | MSC | B2M, ENG, ANPEP, BSG, CD151, CD276, ITGB1, CD44, CD47, CD59, NT5E, CD81, THY1, SLC3A2 |
| 6a* | Osteoblast | RUNX2, COL1A1, SPARC, GJA1, BGLAP |
| 6b* | NP cell | PAX1, LGALS3, CA12, KRT8 |
| 7 | Multipotent stem cell | COL1A1, FOXC2, GJA1, HES4, EGLN1, EGLN2, EGLN3, GAPDH, CRYAB, IRF1, BATF, TCF3 |
| 8 | Proliferating MSC | MME, ENG, VCAM1, KIT, TNFRSF1A, TFRC, NT5E, THY1, CASP3, RRM2, PCNA, HIST1H4C, GMNN, RNASEH2A, MELK, CENPK, TK1, TMEM106C, CDCA5, CKS1B |
| 9 | Hypertrophic chondrocyte | SPP1, VEGFA, MEF2C, MMP13, COL10A1, MMP9 |
| 10 | AF cell | MKX, SCX, TNMD, COL1A1, COL5A1, COL12A1, SFRP2, ADAMTS17 |

*Clusters 6a and 6b were designated based on heterogeneity of gene expression within Cluster 6
