# Supplementary figures and images for: Transcriptional profiling of human cartilage endplate cells identifies novel genes and cell clusters underlying degenerated and non-degenerated phenotypes
Source: Arthritis Res Ther. 2024 Jan 3;26:12. doi: 10.1186/s13075-023-03220-6 (PMC10763221; doi:10.1186/s13075-023-03220-6)

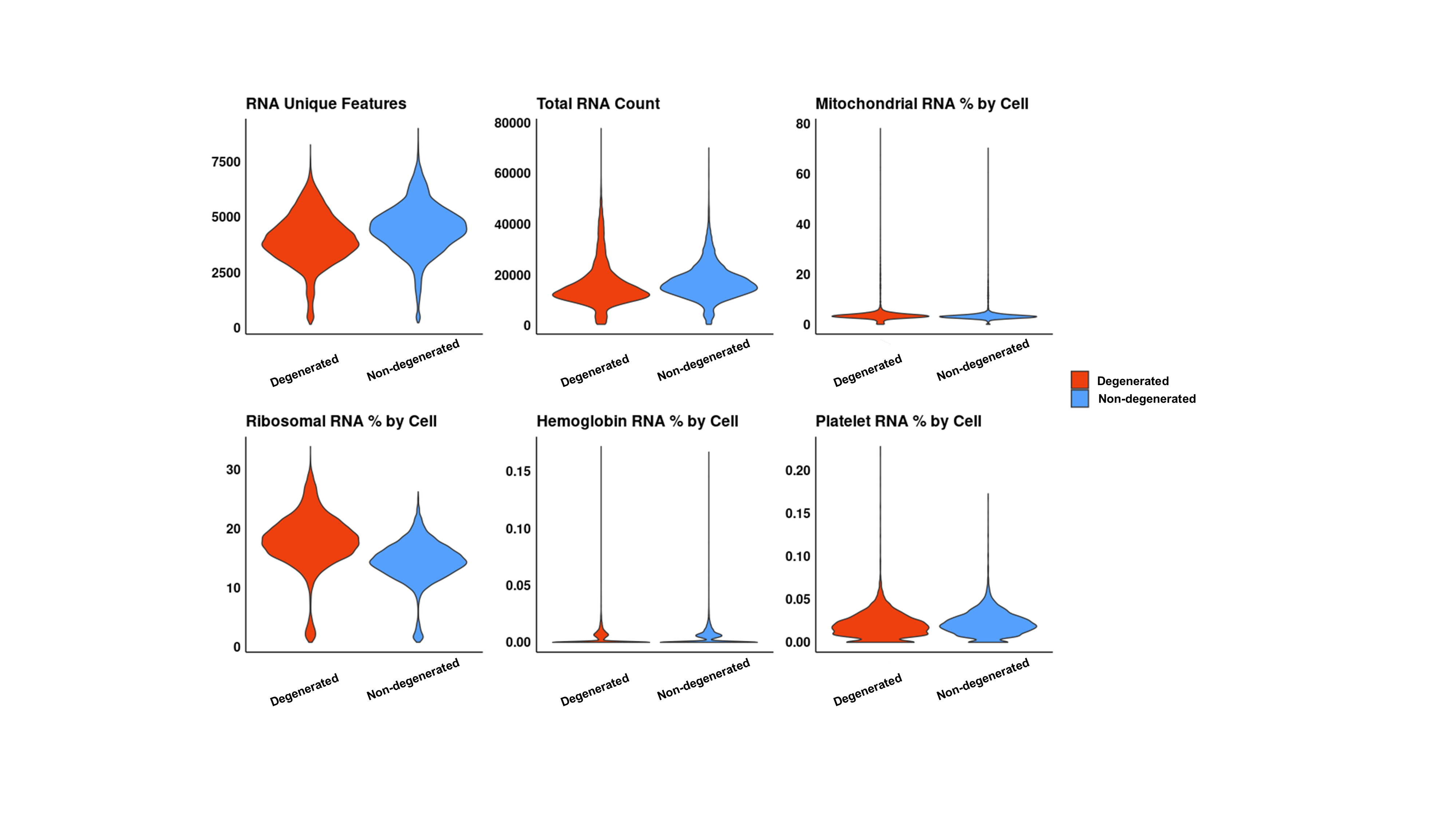

Supplement: Supplementary file 3 — Additional file 3: Figure S1. Pre-filtering quality control. Violin plots showing RNA unique features, total RNA count, mitochondrial RNA content, ribosomal RNA content, hemoglobin RNA content, and platelet RNA content for all cells are shown. [file 13075_2023_3220_MOESM3_ESM.tif]

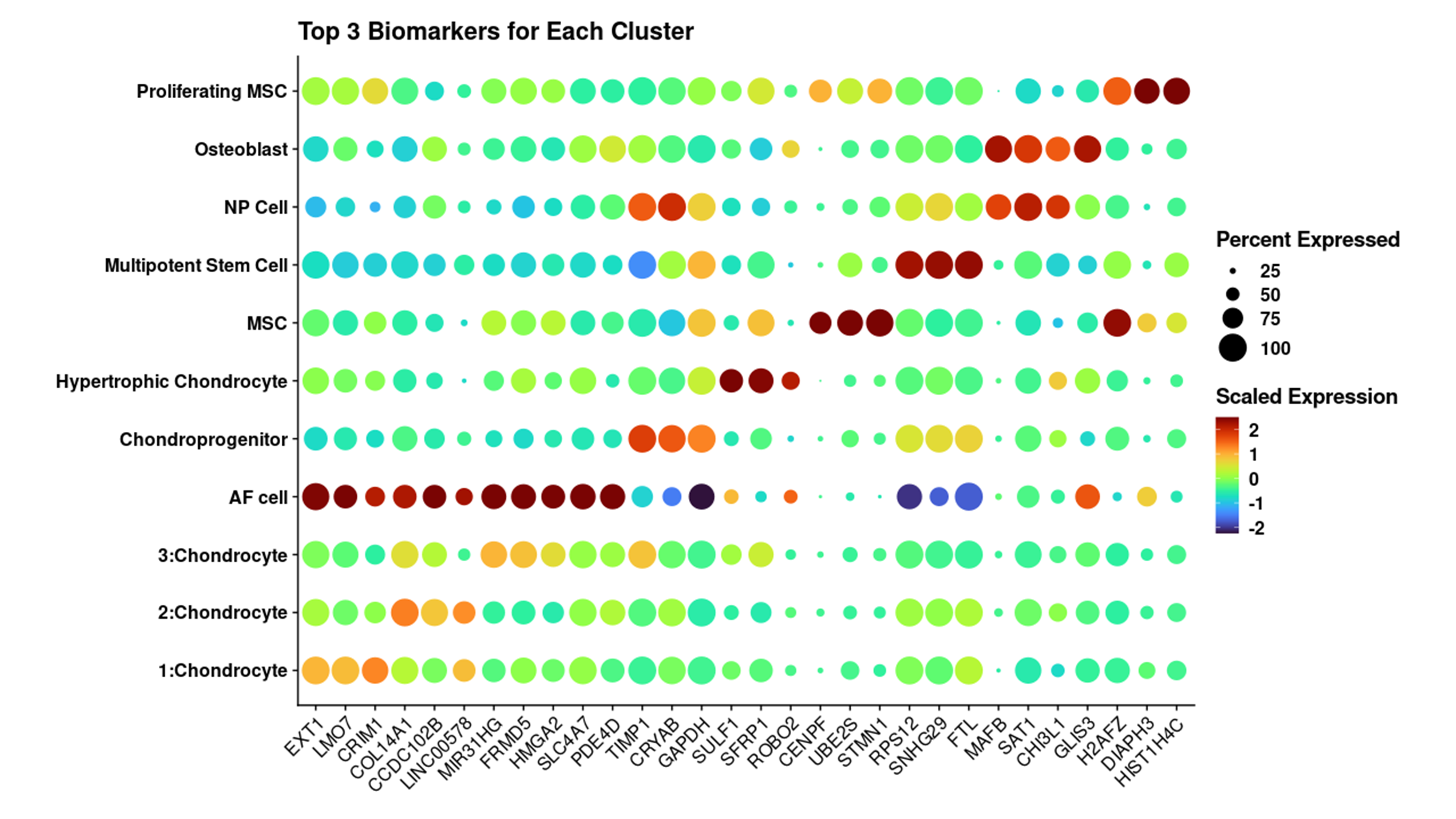

Supplement: Supplementary file 7 — Additional file 7: Figure S2. Dot plot of top 3 markers of each cluster identified in single-cell RNA-Sequencing analysis. [file 13075_2023_3220_MOESM7_ESM.tif]

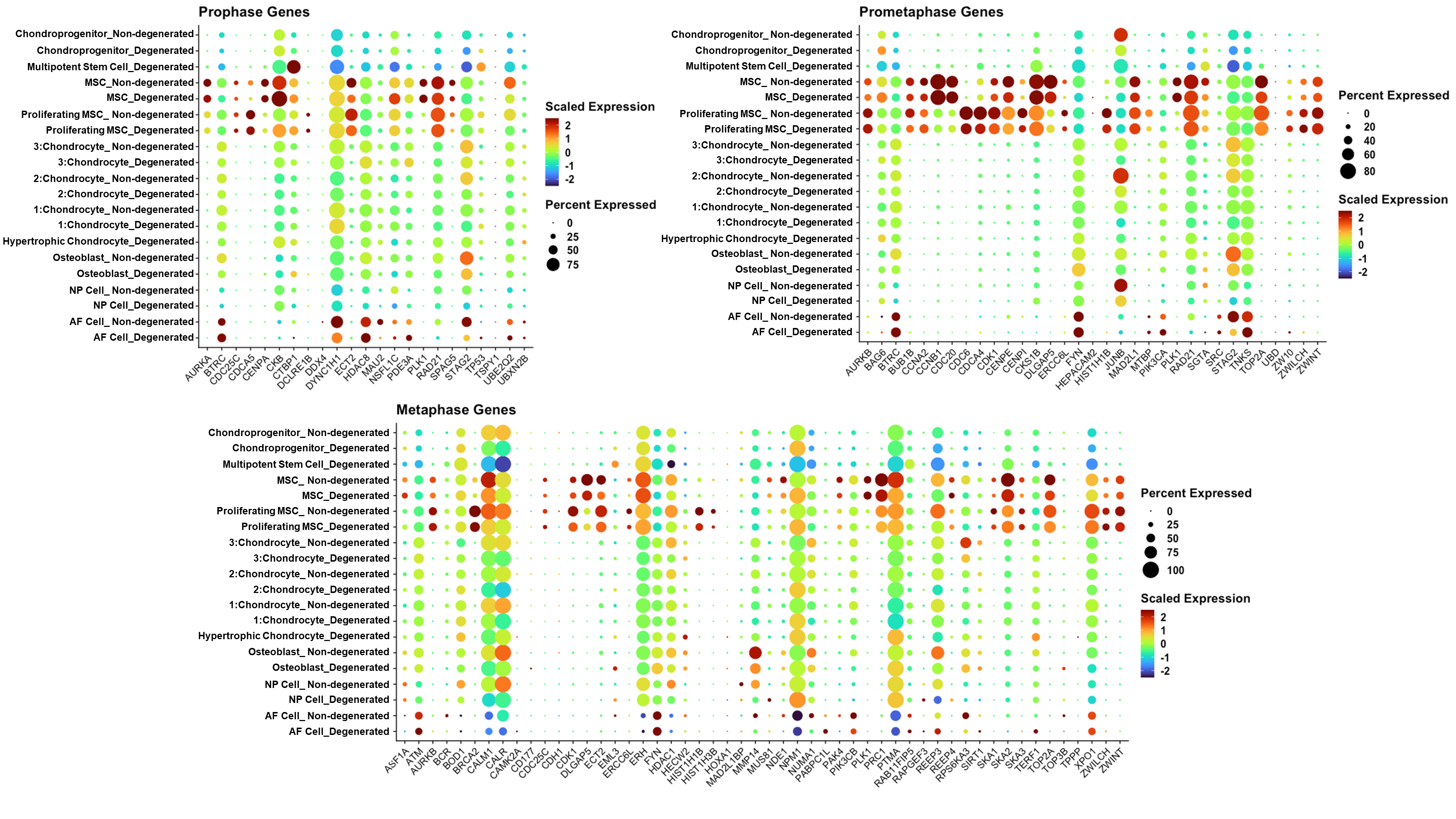

Supplement: Supplementary file 8 — Additional file 8: Figure S3. Dot plots of markers associated with different phases of mitosis. Markers were selected from the following gene sets from https://maayanlab.cloud/Harmonizome/: 1) Prophase (GeneRIF Biological Term Annotations), 2) Prometaphase (GeneRIF Biological Term Annotations), and 3) Metaphase (GeneRIF Biological Term Annotations). [file 13075_2023_3220_MOESM8_ESM.tif]

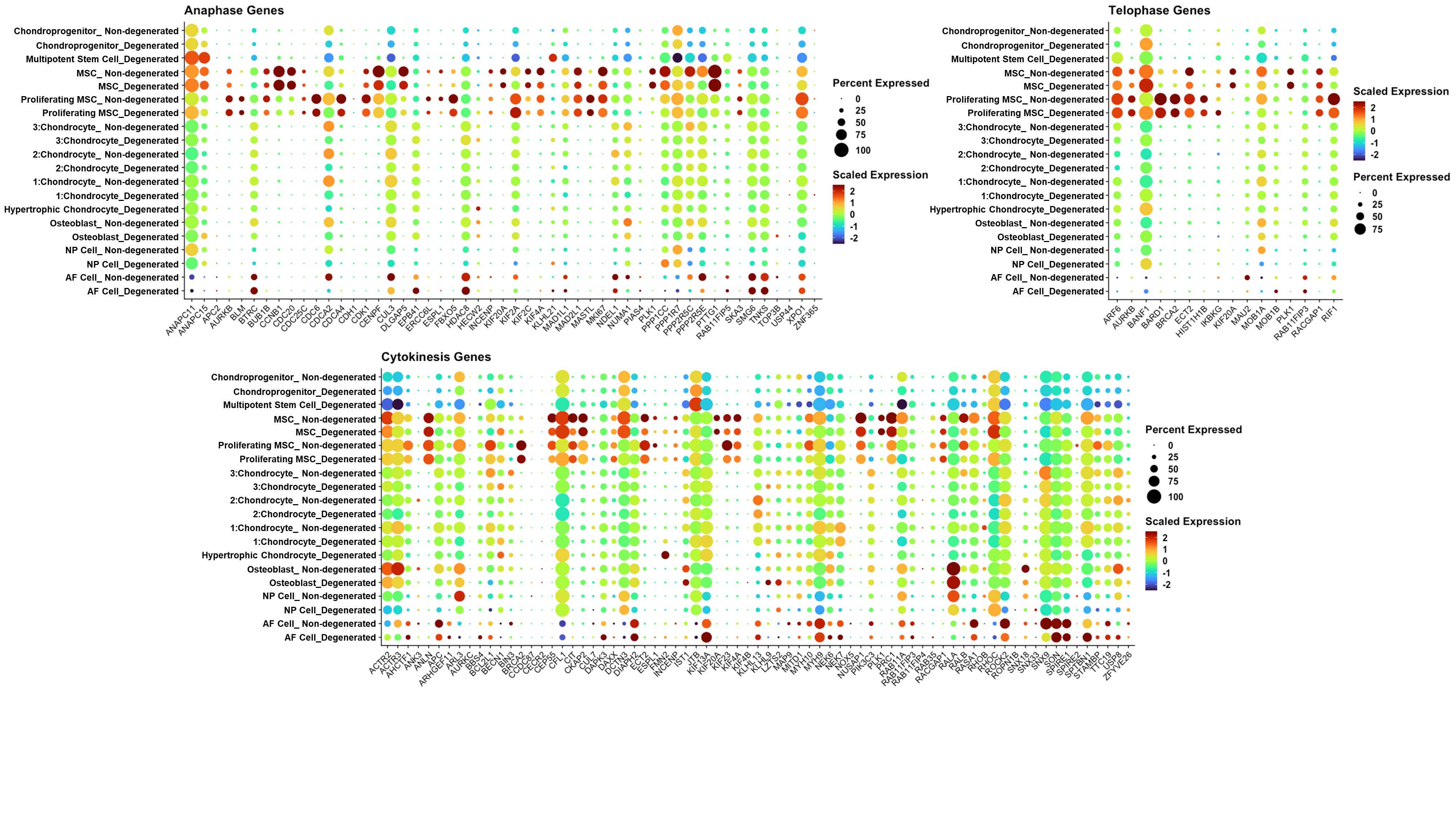

Supplement: Supplementary file 9 — Additional file 9: Figure S4. Dot plots of markers associated with different phases of mitosis. Markers were selected from the following genes sets from https://maayanlab.cloud/Harmonizome/: 1) Anaphase (GeneRIF Biological Term Annotations), 2) Telophase (GeneRIF Biological Term Annotations), and 3) Cytokinesis (GO Biological Process Annotations). [file 13075_2023_3220_MOESM9_ESM.tif]

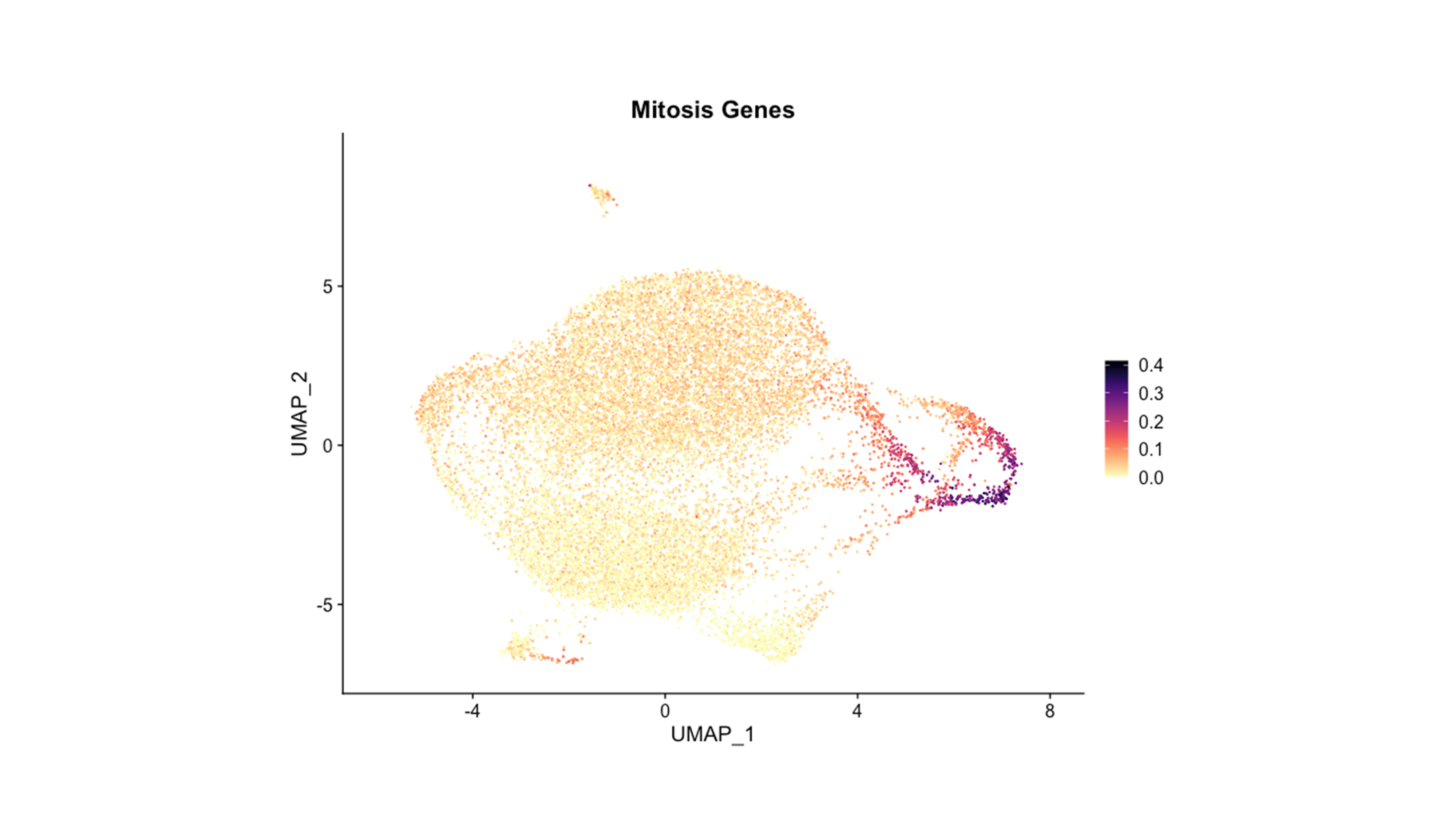

Supplement: Supplementary file 10 — Additional file 10: Figure S5. Module score uMAP of mitosis-related genes from Fig. S3 and Fig. S4. [file 13075_2023_3220_MOESM10_ESM.tif]

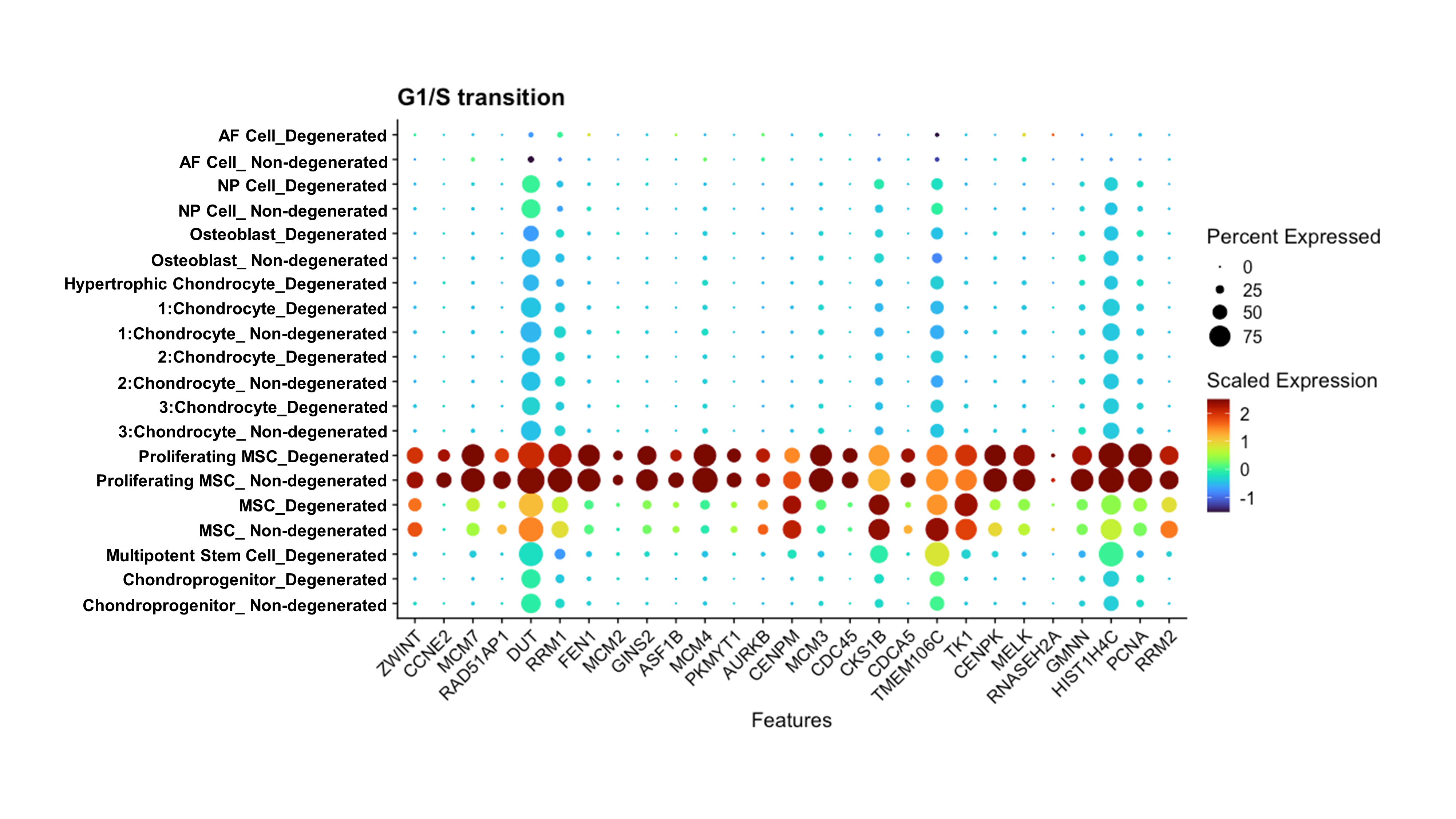

Supplement: Supplementary file 11 — Additional file 11: Figure S6. Dot plot of markers associated with G1/S phase of cell cycle. Markers were selected from gene sets from https://maayanlab.cloud/Harmonizome/. [file 13075_2023_3220_MOESM11_ESM.tif]

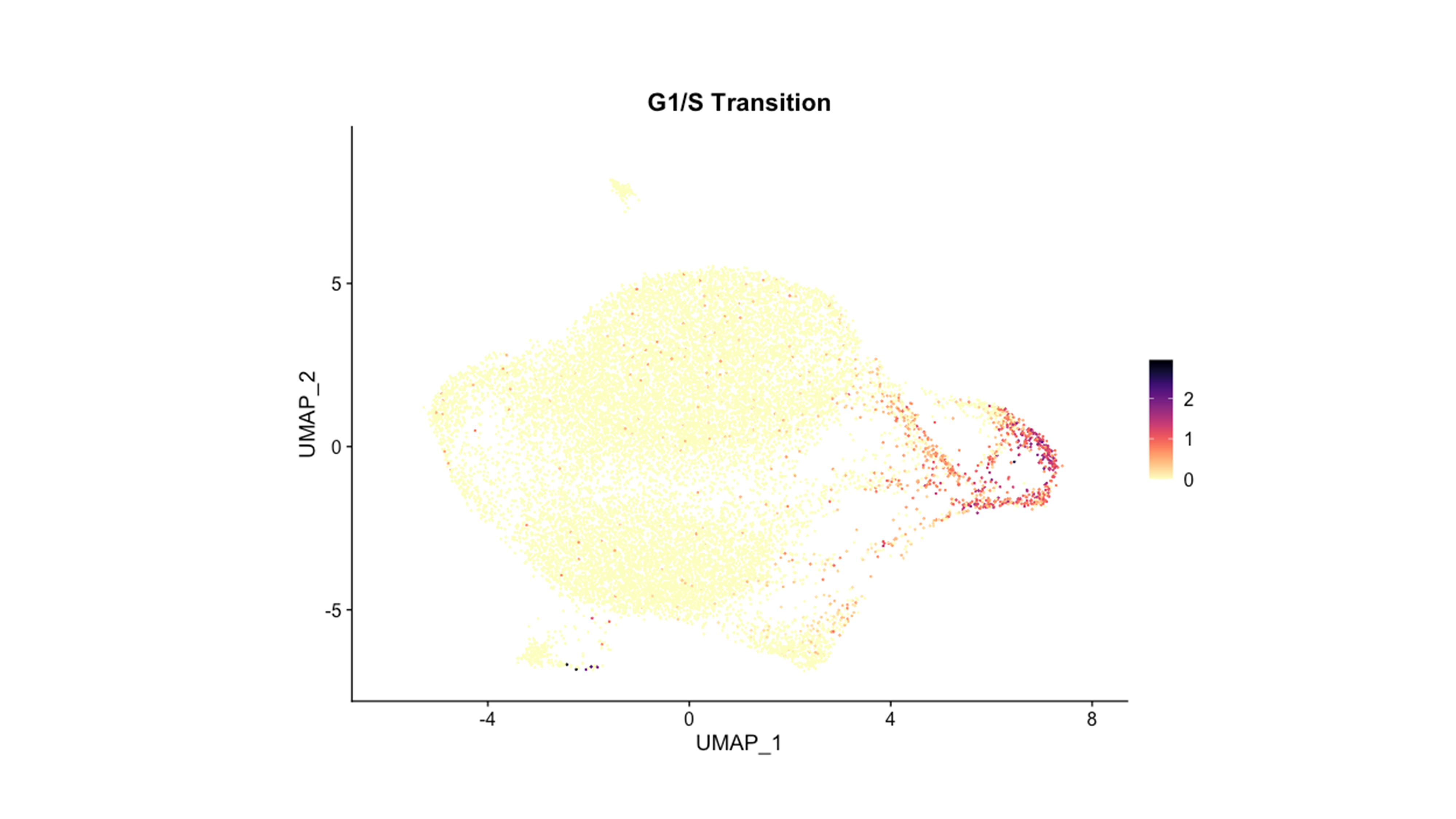

Supplement: Supplementary file 12 — Additional file 12: Figure S7. Module score uMAP of G1/S transition genes from Fig. S6. [file 13075_2023_3220_MOESM12_ESM.tif]

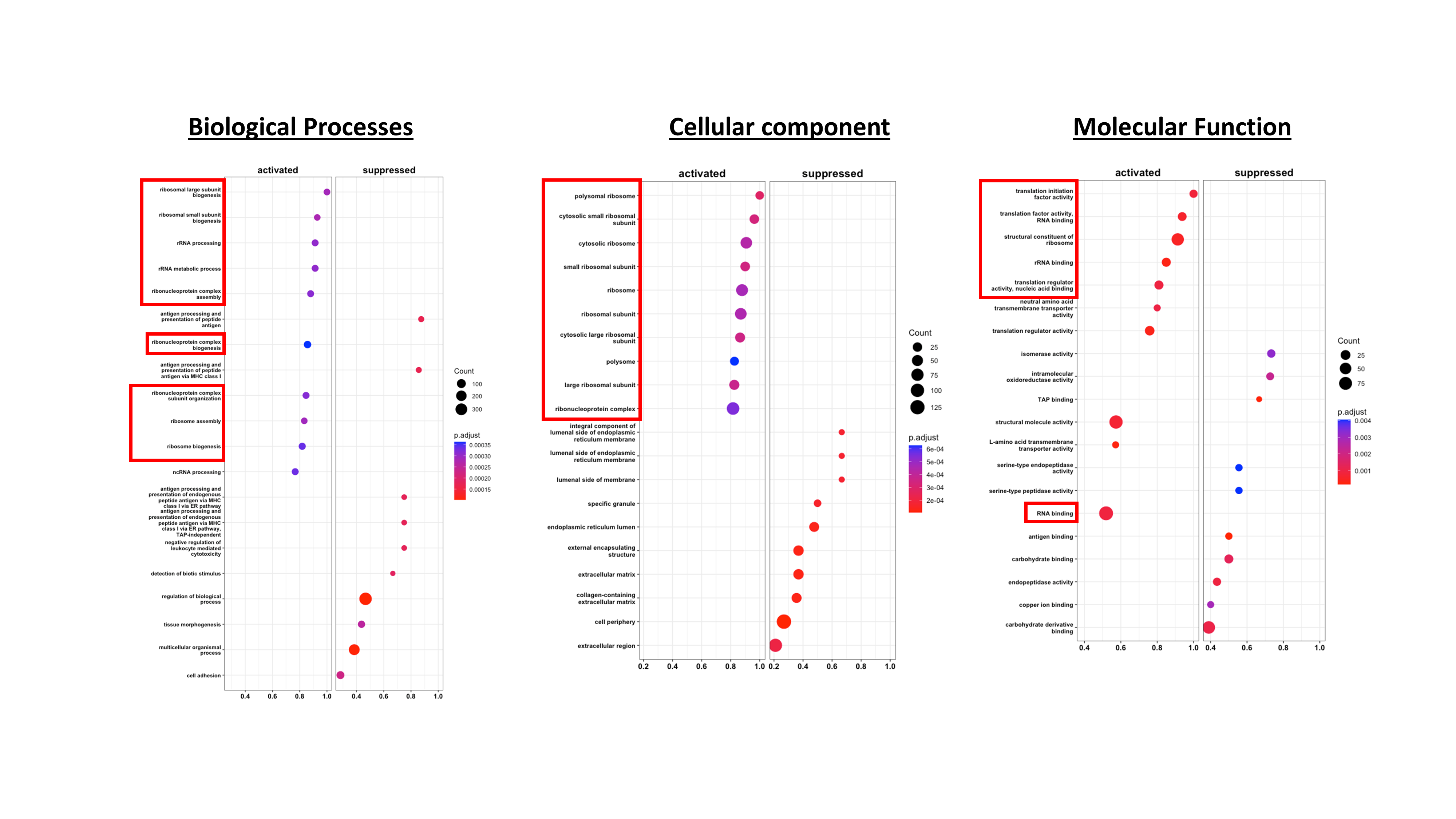

Supplement: Supplementary file 13 — Additional file 13: Figure S8. Gene ontology comparing Non-degenerated and Degenerated Chondrocyte 1. Red boxes indicate pathways associated with ribosomes, protein translation, and mitochondrial function. “Activated” pathways are enriched in the degenerated sample and “Suppressed” pathways are enriched in the non-degenerated sample. [file 13075_2023_3220_MOESM13_ESM.tif]

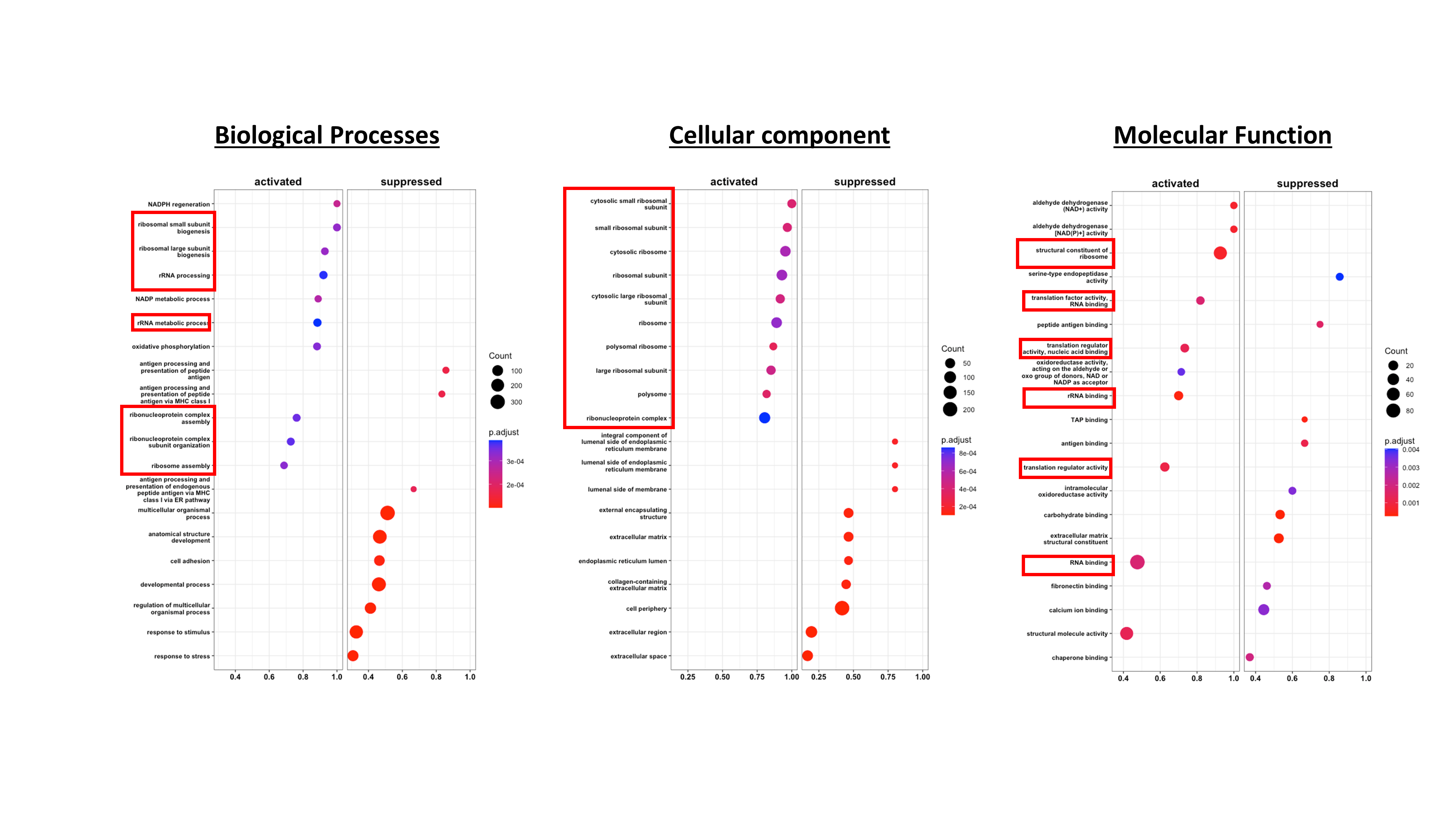

Supplement: Supplementary file 14 — Additional file 14: Figure S9. Gene ontology comparing Non-degenerated and Degenerated Chondrocyte 2. Red boxes indicate pathways associated with ribosomes, protein translation, and mitochondrial function. “Activated” pathways are enriched in the degenerated sample and “Suppressed” pathways are enriched in the non-degenerated sample. [file 13075_2023_3220_MOESM14_ESM.tif]

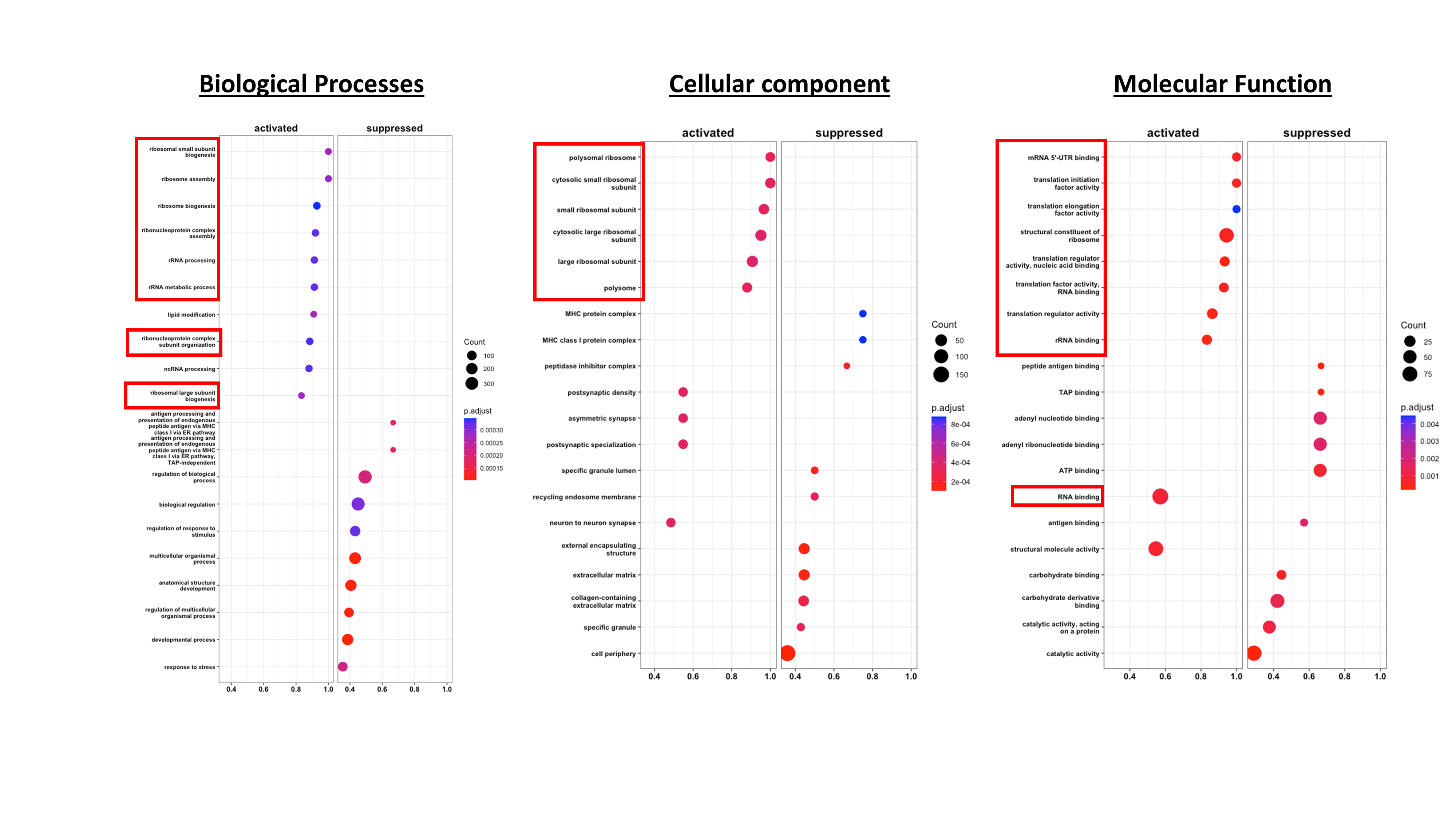

Supplement: Supplementary file 15 — Additional file 15: Figure S10. Gene ontology comparing Non-degenerated and Degenerated Chondrocyte 3. Red boxes indicate pathways associated with ribosomes, protein translation, and mitochondrial function. “Activated” pathways are enriched in the degenerated sample and “Suppressed” pathways are enriched in the non-degenerated sample. [file 13075_2023_3220_MOESM15_ESM.tif]

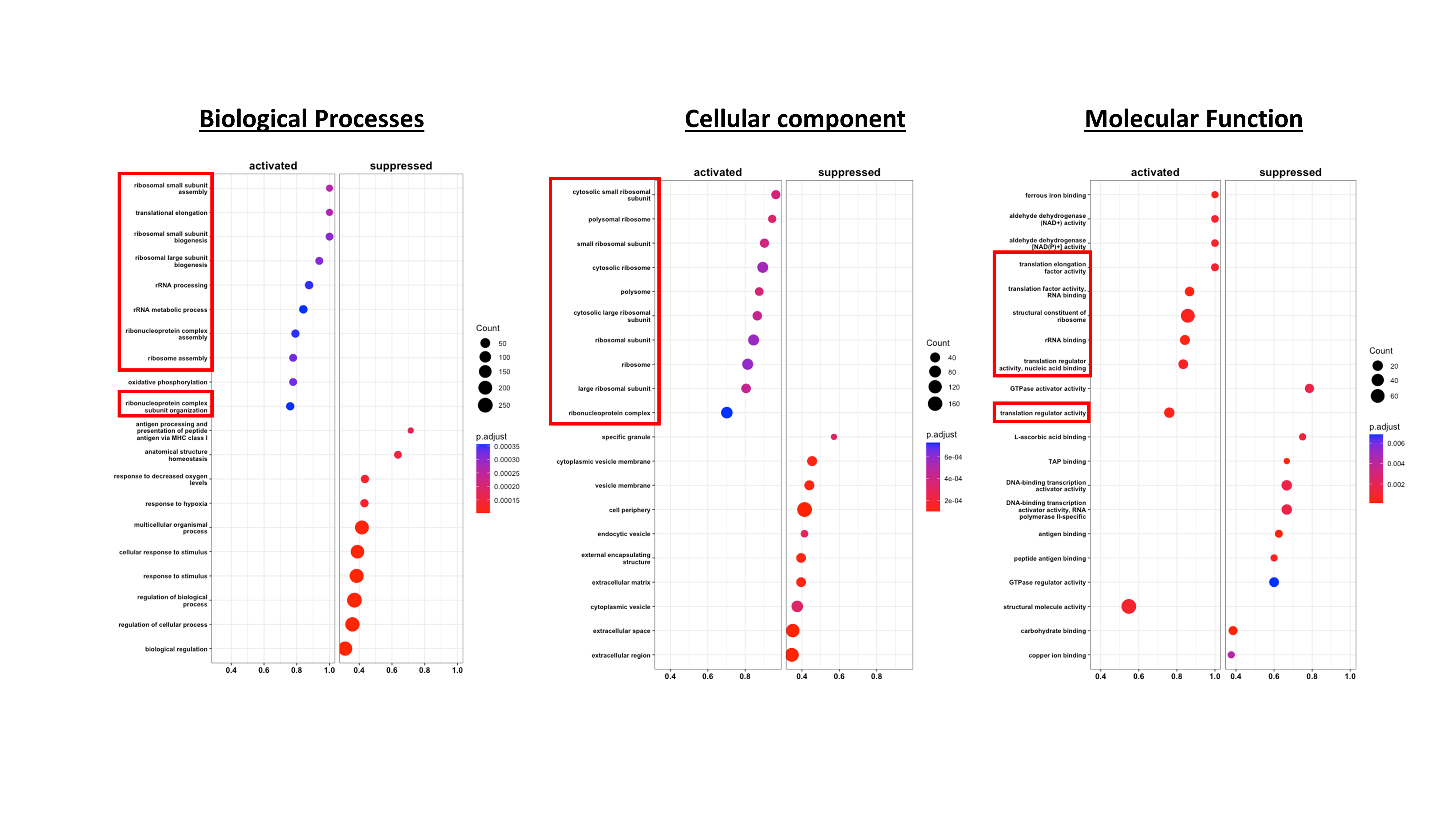

Supplement: Supplementary file 16 — Additional file 16: Figure S11. Gene ontology comparing Non-degenerated and Degenerated Chondroprogenitors. Red boxes indicate pathways associated with ribosomes, protein translation, and mitochondrial function. “Activated” pathways are enriched in the degenerated sample and “Suppressed” pathways are enriched in the non-degenerated sample. [file 13075_2023_3220_MOESM16_ESM.tif]

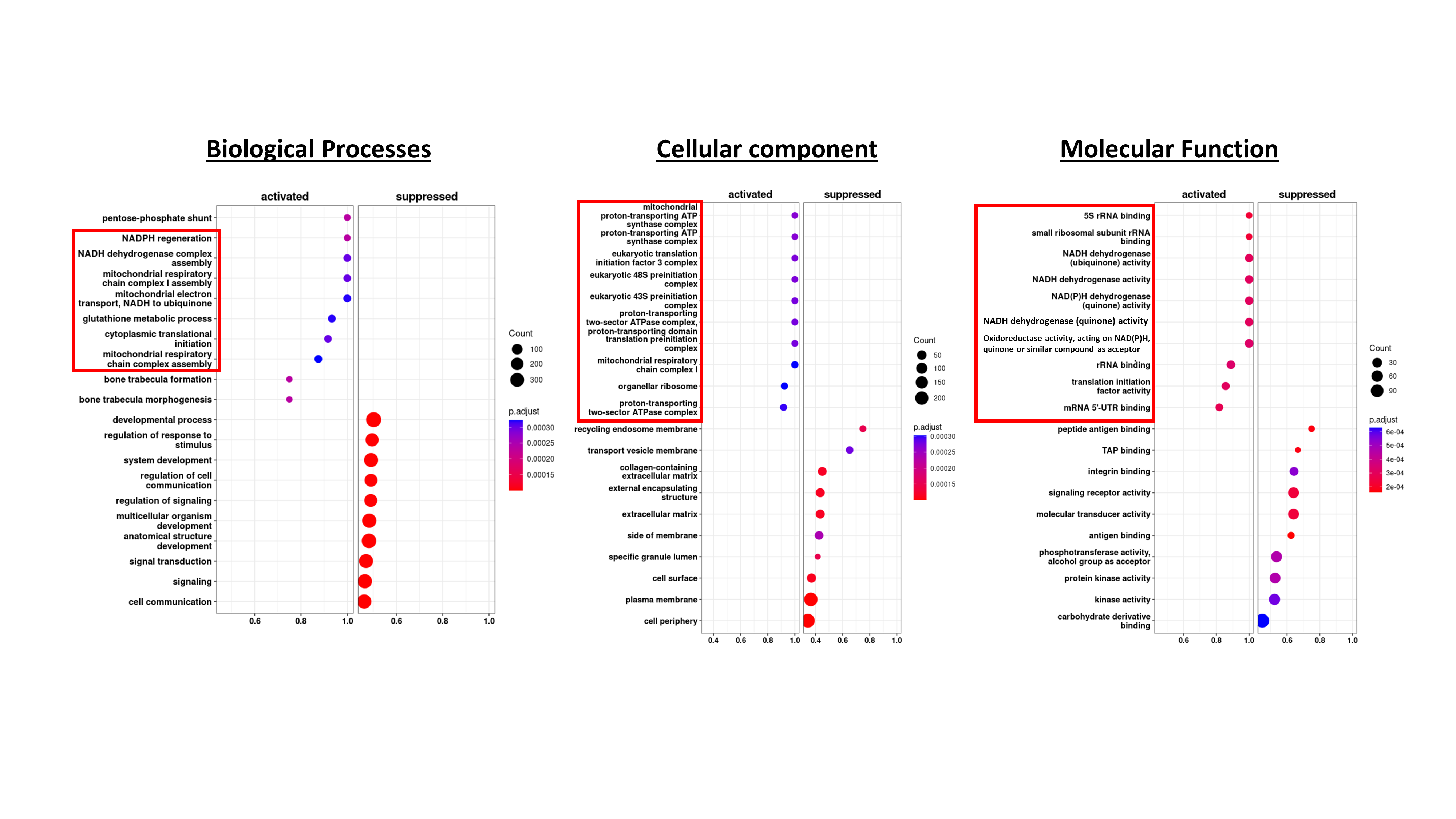

Supplement: Supplementary file 17 — Additional file 17: Figure S12. Gene ontology comparing Non-degenerated and Degenerated MSCs. Red boxes indicate pathways associated with ribosomes, protein translation, and mitochondrial function. “Activated” pathways are enriched in the degenerated sample and “Suppressed” pathways are enriched in the non-degenerated sample. [file 13075_2023_3220_MOESM17_ESM.tif]

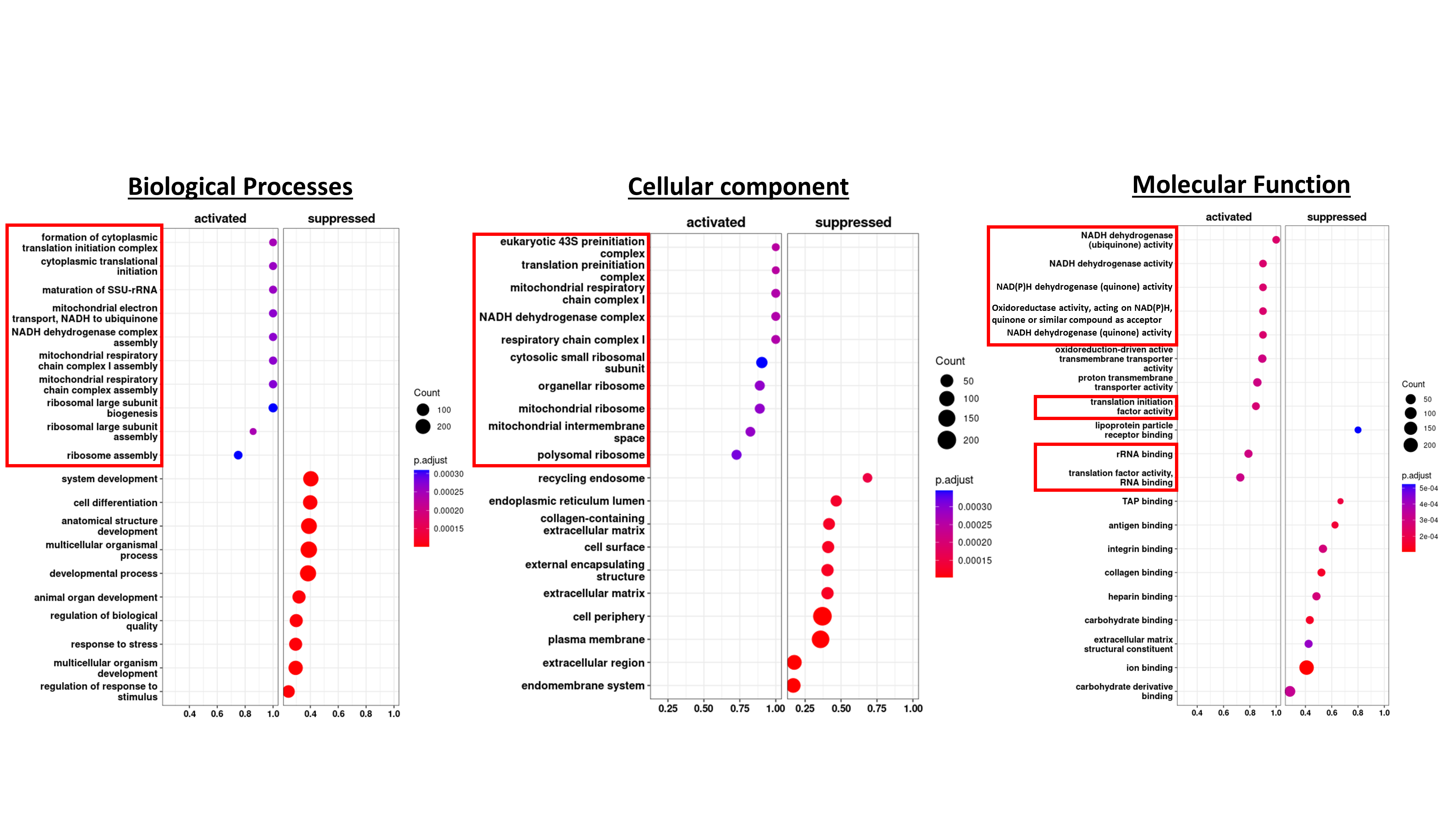

Supplement: Supplementary file 18 — Additional file 18: Figure S13. Gene ontology comparing Non-degenerated and Degenerated Proliferating MSCs. Red boxes indicate pathways associated with ribosomes, protein translation, and mitochondrial function. “Activated” pathways are enriched in the degenerated sample and “Suppressed” pathways are enriched in the non-degenerated sample. [file 13075_2023_3220_MOESM18_ESM.tif]
